# Spatiotemporal Dynamic Assembly/Disassembly of Organelle‐Mimics Based on Intrinsically Disordered Protein‐Polymer Conjugates

**DOI:** 10.1002/advs.202102508

**Published:** 2021-11-01

**Authors:** Hang Zhao, Emmanuel Ibarboure, Vusala Ibrahimova, Ye Xiao, Elisabeth Garanger, Sébastien Lecommandoux

**Affiliations:** ^1^ Univ. Bordeaux CNRS Bordeaux INP LCPO UMR 5629 Pessac F‐33600 France

**Keywords:** aqueous two‐phase systems, elastin‐like polypeptides, liquid–liquid phase separation, microfluidics, peptide–polymer conjugates, synthetic protocells

## Abstract

Design of reversible organelle‐like microcompartments formed by liquid–liquid phase separation in cell‐mimicking entities has significantly advanced the bottom‐up construction of artificial eukaryotic cells. However, organizing the formation of artificial organelle architectures in a spatiotemporal manner within complex primitive compartments remains scarcely explored. In this work, thermoresponsive hybrid polypeptide‐polymer conjugates are rationally engineered and synthesized, resulting from the conjugation of an intrinsically disordered synthetic protein (IDP), namely elastin‐like polypeptide, and synthetic polymers (poly(ethylene glycol) and dextran) that are widely used as macromolecular crowding agents. Cell‐like constructs are built using droplet‐based microfluidics that are filled with such bioconjugates and an artificial cytoplasm system that is composed of specific polymers conjugated to the IDP. The distinct spatial organizations of two polypeptide‐polymer conjugates and the dynamic assembly and disassembly of polypeptide‐polymer coacervate droplets in response to temperature are studied in the cytomimetic protocells. Furthermore, a monoblock IDP with longer length is concurrently included with bioconjugates individually inside cytomimetic compartments. Both bioconjugates exhibit an identical surfactant‐like property, compartmentalizing the monoblock IDP coacervates via temperature control. These findings lay the foundation for developing hierarchically structured synthetic cells with interior organelle‐like structures which could be designed to localize in desired phase‐separated subcompartments.

## Graphical abstract

Two elastin‐like polypeptide‐block‐polymer conjugates as building blocks for artificial organelles are individually encapsulated inside cytomimetic cell‐like microdroplets. Regulation of temperature enables dynamic assembly/disassembly and programmable position of organelle‐mimics in crowded medium, as well as rapid compartmentalization of hydrophobic coacervates from a monoblock ELP.

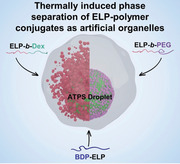

## 1 Introduction

All living eukaryotic cells with structural features of complex and organized intracellular compartments can spatially concentrate components and precisely arrange diverse biochemical processes simultaneously.^[^ ^1^ ^]^ Inspired by this exceptional characteristic of cells, building multicompartmental artificial cell‐like systems that could enrich complex biomimetic functions, has attracted considerable interests in the past decade.^[^ ^2^ ^]^ To date, the design of multicompartmental structures has focused primarily on vesosomes that are essentially membrane‐bound vesicle(s)‐in‐vesicle,^[^ ^3^ ^]^ for example liposome‐in‐liposome,^[^ ^4^ ^]^ liposome‐in‐polymersome,^[^ ^5^ ^]^ polymersome‐in‐polymersome,^[^ ^6^ ^]^ and other systems of which membrane barriers can be constructed by protein–polymer nanoconjugates.^[^ ^7^ ^]^ A decade ago, Brangwynne and co‐workers discovered P granules in embryo, which are membraneless compartments exhibiting liquid‐like behaviors.^[^ ^8^ ^]^ Since then, there has been an increasing body of evidence showing eukaryotic cells contain numerous membraneless compartments.^[^ ^9^ ^]^ Such compartments that lack membranes are assembled through a process of liquid–liquid phase separation (LLPS) in specific areas either cytoplasm or nucleus, that locally concentrate precise biocomponents, mediate biochemical processes spatiotemporally and boost reaction rates greatly.^[^ ^10^ ^]^ Therefore, apart from continuous study on multicompartmental cell‐mimics based upon membranous systems, an intensive research has provided strategies on designing and building such phase‐separated membraneless subcellular compartments within cell mimicking entities, in terms of structuration and functionalities.^[^ ^11^ ^]^ Despite the impressive progress of assembling multicompartmental systems based on the emerging LLPS in artificial biology, crucial challenges remain. Among them, the reversible assembly of organelle‐like architectures with precise space and time control in complex biomimetic compartments would represent a major achievement.

Intrinsically disordered proteins (IDPs) are a class of proteins that lack a defined and ordered 3D structure and possess highly repetitive and low complexity sequences.^[^ ^9^, ^12^ ^]^ These IDPs are discovered to be ubiquitous molecular drivers of intracellular phase separation of forming liquid‐like membraneless bodies. Elastin‐like polypeptides (ELPs), a class of thermo‐responsive bioengineered proteins, have emerged as a remarkable model of IDP, owing to their low sequence complexity and similar biophysical characteristics to IDPs.^[^ ^13^ ^]^ The molecular structure of ELPs is composed of repeat units of a Val‐Pro‐Gly‐Xaa‐Gly pentapeptide sequence, (VPGXG), where the guest residue (X) can be any amino acid with the exception of proline. The hallmark of ELPs is to exhibit lower critical solubility temperature phase transition in water. Below their phase transition temperature, referred as cloud point (*T*_cp_), they are water‐soluble, while upon heating the ELPs above their *T*_cp_ they undergo simple phase separation into an insoluble and dense coacervate phase.^[^ ^14^ ^]^ Recently studies demonstrated that regulation of both guest residue and chain length of ELPs enables programmable assembly of hierarchical core‐shell coacervate‐based artificial organelles in emulsion‐templated protocells,^[^ ^15^ ^]^ and also showed the ELP dynamically and spatially assembled ELP‐rich coacervates in macromolecularly crowded synthetic cellular compartments.^[^ ^11e^ ^]^ Beyond having ELP in artificial chassis, de novo design and expression of artificial amphiphilic ELPs in *Escherichia coli* was programmed to self‐assemble into subcellular compartments in vivo.^[^ ^16^ ^]^ In addition Wang et al. showed that, in living cells, a variety of intracellular polymerization‐triggered nanosystems with different topological structures can be constructed by encoding monomeric ELPs sequences.^[^ ^17^ ^]^ These developments in assembly of ELPs into organelle‐like systems indeed provide insights on reversible formation of subcellular compartments using IDPs both in vitro and in vivo. Nevertheless, the construction of spatially organized artificial organelles in hierarchically structured protocell models has received only limited attention due to the lack of rational design of molecular building blocks for organelle‐like compartments and cell‐like systems. Several research works have sought to develop coacervate‐based organelles that can specifically bind to artificial cell membranes through electrostatic interactions.^[^ ^11f,g^ ^]^ However, these studies have only explored the use of oppositely charged coacervates and membrane building blocks to recruit organelle‐mimics to the membrane and lock their movement.

**Figure 1:**
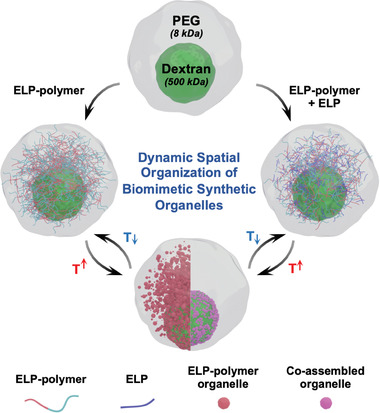
Schematic illustration showing the assembly of a macromolecularly crowded (dextran 500 kDa/PEG 8 kDa) cell‐mimicking construct in which spatially organized ELP‐polymer artificial organelle architectures or co‐assembled assemblies from the monoblock ELP and ELP‐polymer bioconjugates are formed.

In this work, we addressed this unmet need through the engineering of ELP‐polymer conjugates and the use of aqueous two‐phase system (ATPS) as a synthetic cytoplasm (**Figure** **1**). To mimic the macromolecular crowded cellular milieu, a binary polymeric phase that contains dextran and poly(ethylene glycol) (PEG) has been selected to form phase‐separated interior within emulsion‐based cell‐like compartments which were produced at high‐throughput by microfluidics. We synthesized ELP‐polymer bioconjugates, where ELP (artificial IDP) was covalently conjugated to dextran and PEG, respectively. Next, the resulting two types of ELP‐polymer bioconjugates were individually compartmentalized within cytomimetic cellular entities, where partitioning features between dextran/PEG ATPS, and dynamic assembly of organelle‐like structures were studied as function of temperature. We especially demonstrated that above their cloud point temperature (*T*_cp_), both ELP‐polymer conjugates were able to respond to temperature change, phase‐separated and self‐assembled into coacervate‐core micelles, mimicking membraneless organelles (Figure 1). Interestingly, these ELP‐polymer organelle‐like constructs were mostly distributed at the interface of the dextran/PEG phase of the ATPS synthetic cytoplasm. Finally, in order to increase complexity of the system, a monoblock ELP was included into cell‐mimicking chassis together with each ELP‐polymer. By tuning the temperature above the monoblock ELP's *T*_cp_ but below *T*_cp_ of ELP‐polymer, both bioconjugates were shown to serve as surfactants that are capable to stabilize and sequester encapsulants of coacervates formed by phase separation of the monoblock ELP, and to migrate to the aqueous/aqueous interface of dextran/PEG (Figure 1). We believe these findings are expected to facilitate developments of synthetic organelles/cells in a bottom‐up fashion allowing precise and dynamic control over localization of complex multi‐components.

## 2 Results and Discussion

### 2.1 Synthesis of ELP‐Polymer Bioconjugates and their Partitioning Properties between Two Polymeric Phases within Cytomimetic Protocells

In a first step, we examined the partitioning property of the IDP model within macromolecularly crowded ATPS compartments. We specifically focused on an ELP containing 40 repeat units of a pentapeptide presenting Val/Met as guest residue in a 3:1 ratio, namely ELP[M_1_V_3_‐40] as previously reported.^[^ ^18^ ^]^ This ELP was produced recombinantly in *E. coli* bacteria and purified by inverse transition cycling (see details in the Experimental Section).^[^ ^14a^ ^]^ To monitor the partition event inside cell‐like compartments, the model ELP was fluorescently labeled with a rhodamine dye (Scheme S1; Figures S1 and S2, Supporting Information). Based on our previous work concerning the behavior of artificial IDP in macromolecular agents encapsulated protocells,^[^ ^11e^ ^]^ we injected in a glass capillary‐based microfluidic device the rhodamine‐labeled ELP (0.5 mg mL^−1^) along with the dextran phase (8 wt%) and the PEG solution (16 wt%) in two separate channels at the same flow rate. These two aqueous phases met at the orifice of the injection tube and were pinched‐off by an oil phase (TEGOSOFT DEC/mineral oil) with a non‐ionic surfactant (ABIL EM 90), forming paired water‐in‐oil microdroplets (Figure S3, Supporting Information). In the resulting microdroplets, concentrations of all solutes were therefore diluted twice leading to dextran (4 wt%) and PEG (8 wt%) for the ATPS system, which displayed two‐phase states at both 10 and 50 °C (Figure S4, Supporting Information). Immediately after collection of emulsion droplets from the device, the rapid partition of the rhodamine‐labeled ELP[M_1_V_3_‐40] into the PEG‐rich phase was observed, which is in a good arrangement with our previous findings showing that below the transition temperature, hydrated ELPs with coil‐like chain structures prefer to enrich in the PEG phase, regardless of the length of the artificial IDPs.^[^ ^11e^ ^]^

**Figure 2:**
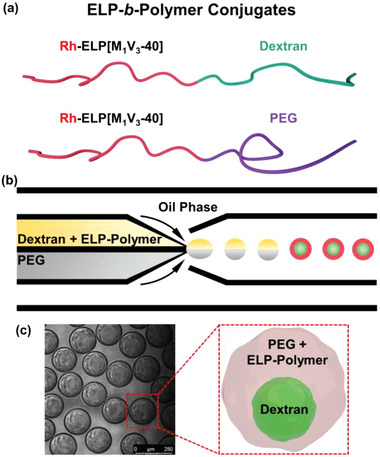
Molecular engineering of ELP[M_1_V_3_‐40] with dextran and PEG biomacromolecules, respectively, and encapsulation of bioconjugates inside cytomimetic compartments. a) Schemes of conjugates of ELP‐*b*‐Dex (dextran 8 kDa) and ELP‐*b*‐PEG (methoxypolyethylene glycol 2 kDa) upon covalent conjugation. The weight average molecular weight (*M*_w_) for ELP‐*b*‐Dex is 24.9 kDa and ELP‐*b*‐PEG is 19.4 kDa, respectively. b) The graphic illustration of microfluidic preparation of water‐in‐oil emulsion‐based protocellular entities in which ELP‐*b*‐Dex or ELP‐*b*‐PEG is individually encapsulated together with the dextran/PEG system; here, the upper phase is dextran and ELP‐polymer conjugate, and the lower phase is PEG. c) Schematic and optical image of as‐formed microdroplets containing the artificial cytoplasm and ELP‐polymer conjugates that phase separate rapidly after collection from the microfluidic device. Green is FITC‐dextran and red is rhodamine‐labeled ELP‐polymer bioconjugates.

In order to precisely control the localization of our thermo‐sensitive IDP analogue in one of the phases or at the interface of the ATPS system, we molecularly engineered our ELP. Bioconjugates combining ELP with either dextran or PEG, namely ELP[M_1_V_3_‐40]‐*b*‐dextran (ELP‐*b*‐Dex) and ELP[M_1_V_3_‐40]‐*b*‐PEG (ELP‐*b*‐PEG), respectively, were synthesized based on a coupling reaction between ELP[M_1_V_3_‐40] functionalized at the *N*‐terminal end with an alkyne group and azido‐terminated biomacromolecules (dextran or PEG) through chemoselective copper(I)‐catalyzed azide‐alkyne cycloaddition (CuAAC) reaction (**Figure** **2**a; Scheme S2 and Figures S5, S6, and S7, Supporting Information).^[^ ^18^, ^19^ ^]^ Both ELP‐polymer conjugates were also fluorescently labeled with rhodamine to enable visualization of their spatial distribution within cytomimetic compartments (Schemes S3 and S4; Figure S8, Supporting Information). To construct the cytomimetic assemblies with ELP‐polymer conjugates encapsulated, either ELP‐*b*‐Dex or ELP‐*b*‐PEG (0.25 mg mL^−1^) replaced the ELP, and the paired microdroplets were produced in the same manner as aforementioned microfluidic approach to assess preferential partitioning of rhodamine‐labeled ELP, as shown in Figure 2b (see the Experimental Section). The highly monodisperse paired water‐in‐oil emulsions passed through the collection capillary and were collected and imaged on a glass slide that was sealed with a cover slip (Figure 2c). LLPS between the dextran/PEG artificial cytoplasm occurred rapidly, forming a dispersed dextran‐rich phase and a surrounding PEG phase as a result of the selected ratio between dextran (4 wt%) and PEG (8 wt%) where PEG plays a role as majority phase.

**Figure 3:**
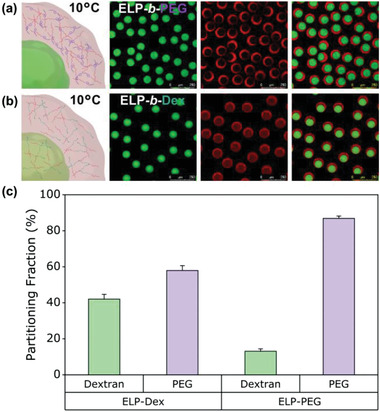
Distinct partitioning behaviors of ELP‐*b*‐PEG and ELP‐*b*‐Dex at 10 °C inside cytomimetic protocells where dextran and PEG phase separate into water‐in‐water state that is predominantly driven by repulsive interactions between binary macromolecules. a) Schematic representation and representative confocal images of spatial position of the ELP‐*b*‐PEG within microdroplets. Green is FITC‐dextran and red is Rh‐ELP‐*b*‐PEG. b) Schematic illustration and representative confocal images show the distribution of the ELP‐*b*‐Dex inside microdroplets. Green is FITC‐dextran and red is Rh‐ELP‐*b*‐Dex. c) The bar graphs show inside cell‐like constructs, different partitioning properties of the ELP‐*b*‐PEG (*n* = 33) and the ELP‐*b*‐Dex (*n* = 39) between dextran‐rich and PEG‐rich phases, respectively. Error bars represent the standard deviation. Care was taken to keep similar parameters when imaging the ELP‐polymer bioconjugates so as to compare each partitioning performance according to fluorescence signal between the dextran and the PEG phases. Scale bars denote 250 µm.

We then studied the partitioning ability of each bioconjugate in this artificial cytomimetic system. The emulsion‐based compartments in which two ELP‐polymer bioconjugates individually sequestered, were imaged at 10 °C, below the transition temperatures of both bioconjugates. **Figure** **3**a shows a sketch and confocal images of ELP‐*b*‐PEG‐containing microdroplets where fluorescein‐labeled dextran (green) phase separated from the PEG‐rich phase. As visible in the image of red channel, the rhodamine‐labeled ELP‐*b*‐PEG conjugate exhibited significantly uneven spatial organization between the two macromolecular phases with remarkable rhodamine signal detected in the surrounding PEG‐rich phase. This clearly indicated that ELP‐*b*‐PEG conjugate preferentially partitioned into the PEG region. We reasoned that this high level of localization of the ELP‐*b*‐PEG toward the PEG phase could be attributed to the specific affinity of the PEG block of the conjugate to the PEG domain combined with the preferred partitioning of hydrated ELP at 10 °C (below the *T*_cp_).^[^ ^11e^ ^]^ Oppositely, the distribution of the ELP‐*b*‐Dex between the dextran core and PEG‐rich shell of microdroplets was more balanced (Figure 3b), certainly due to the favorable interactions between the dextran block of the bioconjugate and the dextran phase. We experimentally validated this observation by initially mixing the ELP‐*b*‐Dex conjugate with PEG solution (instead of dextran) to produce protocellular entities at the same temperature (10 °C). This resulted in a similar spatial localization of the ELP‐*b*‐Dex between the two phases (Figure S9, Supporting Information). Moreover, it can be seen that demixing between dextran/PEG system still arose rapidly, indicating the phase separation of the macromolecular crowding agents was influenced neither by the different ELP‐polymer conjugates nor by their distinct spatial distribution. Further partitioning of both ELP‐polymer conjugates within these phase‐separated cell‐mimicking droplets was quantified by using confocal fluorescence microscopy images as depicted in Figure 3c. We measured a partitioning fraction for both ELP‐polymer conjugates, which is defined as the fluorescence intensity of each bioconjugate in the dextran/PEG phase divided by its overall fluorescence intensity within the microcompartments. The partitioning fraction of the ELP‐*b*‐PEG toward the PEG phase was found to be 86.9 ± 1.3, whereas fractional value for the ELP‐*b*‐Dex within the PEG‐rich region was 57.9 ± 2.7, thus quantitatively confirming the visual observations. This large difference in partitioning between two types of ELP‐polymer conjugates could quantitatively support that engineering of the ELP through covalent conjugation of macromolecules (dextran or PEG) allowed spatially programmable positioning of conjugates in dextran/PEG cytoplasmic system.

### 2.2 Thermo‐Induced Assembly/Disassembly of Spatially Organized Synthetic Organelle‐Mimics from ELP‐Polymer Bioconjugates

**Figure 4:**
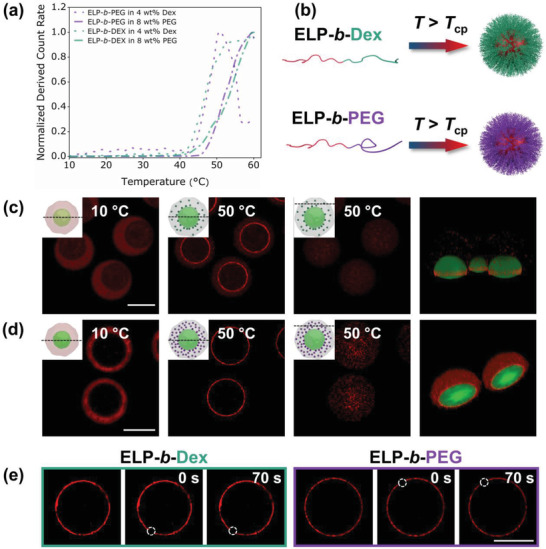
Phase transition of ELP‐polymer bioconjugates. a) Determination of transition temperatures from DLS measurement of both ELP‐polymer conjugates (0.125 mg mL^−1^) in 4 wt% dextran and 8 wt% PEG polymeric phases with operating temperature ranging from 10 to 60 °C. ELP‐*b*‐PEG in dextran phase (purple dots); ELP‐*b*‐PEG in PEG phase (purple dash dots); ELP‐*b*‐Dex in dextran phase (green dots); ELP‐*b*‐Dex in PEG phase (green dash dots). b) Sketch of the assembly of phase separation of both ELP‐polymer into coacervate‐core micelles at the temperature well above their *T*_cp_. c,d) Schemes and representative confocal images of processes of phase transition of ELP‐polymer conjugates when heated to 50 °C, and also 3D reconstruction of droplets from equator of dextran lumens to the top of droplets in both green and red channel, representing FITC‐labeled dextran and rhodamine‐labeled ELP‐polymer conjugates, respectively; (inset) Black dash lines on the side‐view schemes demonstrate the position of focal planes when cytomimetic protocells are imaged. Scale bars denote 75 µm. c) ELP‐*b*‐Dex; d) ELP‐*b*‐PEG. e) FRAP experiments show no recovery for coacervate‐core micelles formed either by ELP‐*b*‐Dex or ELP‐*b*‐PEG at the interface of binary polymeric phases at 50 °C. The bleached areas are highlighted in white dash circles of 10 µm in diameter. Scale bar denotes 50 µm.

We then determined the transition temperatures of the resulting two ELP‐polymer conjugates at 0.125 mg mL^−1^ in each polymer phase (4 wt% dextran or 8 wt% PEG; see the Supporting Information for experimental details). As evidenced by light scattering experiments (**Figure** **4**a), ELP‐*b*‐PEG and ELP‐*b*‐Dex exhibited a phase transition at 43 and 46 °C, respectively, in these conditions, highlighting that molecularly engineered ELP‐polymer conjugates could self‐assemble when increasing temperature, associated to the thermo‐sensitive properties of the ELP. Indeed, when heating above *T*_cp_, the ELP block of the ELP‐polymer conjugate phase separated forming liquid‐like coacervate core, while the hydrophilic dextran or PEG blocks were forming a stabilizing polymer‐corona (Figure 4b). The resulting structures mimicking subcellular organelles were thus named coacervate‐core micelles by adopting the definition from Cohen Stuart.^[^ ^20^ ^]^ To assess the heat‐triggered phase separation and organelle formation of ELP‐polymer conjugates, we individually encapsulated the two fluorescently labeled ELP‐polymer conjugates within cytomimetic microdroplets using the abovementioned microfluidic approach, and heated the microdroplets at 50 °C above the ELP *T*_cp_. Both ELP‐polymer conjugates underwent phase transition by dehydration of the ELP chains to form organelle‐like structures (ELP‐*b*‐Dex Figure 4c; ELP‐*b*‐PEG Figure 4d). In order to better track the phase separation mechanism in 3D, we first fixed the position of imaging focal plane at the equatorial interface of dextran/PEG system. As soon as temperature reached *T*_cp_, the formed coacervate‐core micelles rapidly migrated to the interface. To assess their global distribution, we then moved the focal plane upward to image the top of microdroplets occupied by the PEG‐rich phase since the density of PEG is lower than that of the dextran phase,^[^ ^21^ ^]^ driving the dextran lumen off‐center to the bottom of microdroplets. Interestingly, a large number of ELP‐*b*‐PEG micelles were clearly observed (Figure 4d), and yet ELP‐*b*‐Dex assemblies were barely seen in this PEG‐rich environment (Figure 4c). In addition, subsequent 3D reconstructions from confocal images were built (Figure 4c,d), confirming this inhomogeneous distribution. The particularly strong accumulation of ELP‐*b*‐PEG organelles in the PEG phase confirmed that spatial organization of organelle‐like assemblies at the desired location could be programmed using our molecular‐tailored ELP‐*b*‐PEG. On the contrary, ELP‐*b*‐Dex constructs did not exhibit a pronounced concentration into any phase of the dextran/PEG system. However, notable fluorescence signal was detected at the interface between the two aqueous macromolecular phases, consistent with the 3D reconstructions (Figure 4c). We reasoned that this specific interfacial localization of ELP‐*b*‐Dex assemblies could be attributed to a synergistic effect coming from the surface tension between dextran/PEG system and the density mismatch of ELP‐*b*‐Dex compartments and PEG‐rich phase, as confirmed experimentally (Figure S10, Supporting Information). Next, cooling the ELP‐polymer organelles containing microdroplets back to 10 °C below the bioconjugates’ *T*_cp_ led to a rapid dissolution of the ELP‐polymer assemblies and reversion of each ELP‐polymer bioconjugate to its original partitioning behavior in the dextran/PEG system, evidencing the fully reversible character of the process (Figure S11, Supporting Information). We then studied the dynamic behavior of the interfacial layer of phase‐separated ELP‐polymers. Fluorescence recovery after photobleaching (FRAP) experiments were conducted to determine the fluidity of ELP‐polymer counterparts at the interface between dextran‐rich and PEG‐rich phases (see the Experimental Section). Photobleaching a circular section (10 µm in diameter) of the interfacial layer showed essentially no recovery of rhodamine fluorescence after 70 s, indicating that interface‐enriched ELP‐polymer assemblies were fairly immobile (Figure 4e). This arrested character for both interfacial accumulation of ELP‐polymer entities might result from jamming states of close‐packed assemblies at the interface, leading to the restricted motion of ELP‐polymer coacervate‐core micelles to recover the fluorescence intensity.^[^ ^22^ ^]^ As such, our genetically engineered diblock ELPs with tunable hydrophobicity resembled synthetic block copolymers capable of limiting the coarsening of phase‐separated protein coacervates.^[^ ^15^, ^23^ ^]^

### 2.3 Increased Complexity of Cytomimetic Compartments via Incorporation of a Monoblock ELP

**Figure 5:**
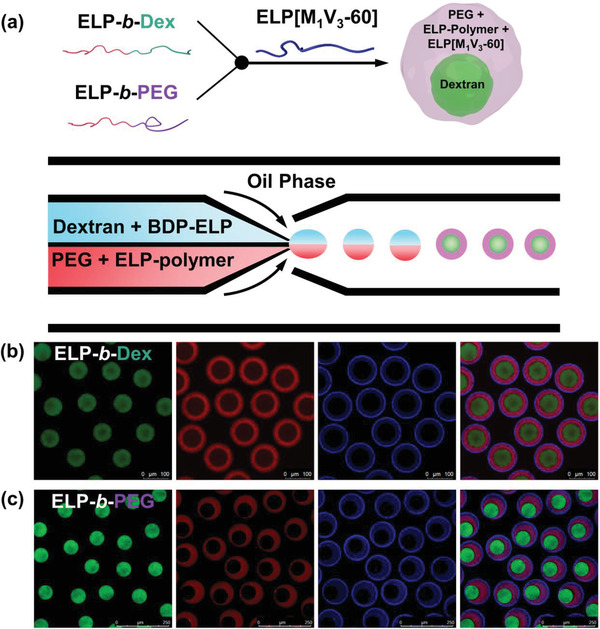
Co‐encapsulation of BODIPY‐labeled monoblock ELP[M_1_V_3_‐60] and individual ELP‐polymer bioconjugates inside cytomimetic protocells. a) Illustration of sequestration of each bioconjugate and monoblock ELP inside a microdroplet and their preferential partitioning between dextran and PEG phases; Scheme of the microfluidic device with separate loadings of two solution mixtures in *θ*‐shaped capillary for producing complex cytomimetic compartments. b,c) Representative confocal microscopy images of ELP‐polymer conjugates, ELP[M_1_V_3_‐60] and ATPS within microdroplets at 10 °C showing spatial organization of ELP‐polymers and monoblock ELP: b) ELP‐*b*‐Dex; c) ELP‐*b*‐PEG. Green is FITC‐dextran, red is either ELP‐*b*‐Dex or ELP‐*b*‐PEG and blue is BDP‐ELP.

After designing and studying the dynamic formation and the favorable positioning of organelle‐like systems from ELP‐polymer bioconjugates, we further explored their ability to compartmentalize hydrophobic components within cytomimetic artificial cells. To evaluate the potential of both ELP‐*b*‐Dex and ELP‐*b*‐PEG bioconjugates, a monoblock ELP with longer length and labeled with BODIPY dye, BDP‐ELP[M_1_V_3_‐60] was selected. We started with two solution mixtures—one is 8 wt% dextran and 0.5 mg mL^−1^ BDP‐ELP, the other is 16 wt% PEG and 0.25 mg mL^−1^ ELP‐polymer, separately loaded into two channels of the theta‐shaped injection capillary (**Figure** **5**a). The monoblock BDP‐ELP[M_1_V_3_‐60] and ELP‐polymers containing protocell droplets were then produced in the same microfluidic system. Microdroplets were collected and immediately imaged at 10 °C below both ELP *T*_cp_ (30 °C) and ELP‐polymer *T*_cp_ (≈45 °C). As expected, the monoblock ELP rapidly partitioned into the PEG‐rich phase because of similar conformation between the coil‐like chain of PEG and the hydrated chain‐like ELP at 10 °C, in agreement with our previous finding,^[^ ^11e^ ^]^ and ELP‐polymer bioconjugates were localized in a similar manner as described earlier (Figure 5b,c).

**Figure 6:**
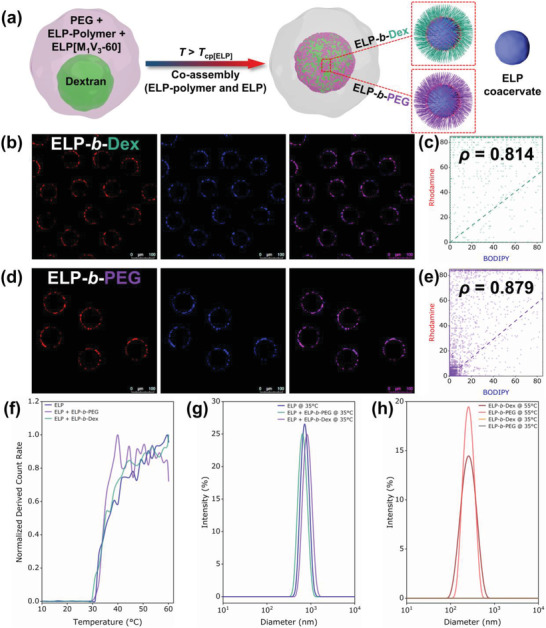
Phase transition behavior performance and spatial redistribution of ELP‐rich assemblies stabilized by ELP‐polymers within protocell droplets. a) Scheme of co‐assembly of the ELP and each ELP‐polymer at 35 °C, and simultaneous migration to the dextran/PEG interface. b) Fluorescence colocalization imaging of co‐assembled ELP‐*b*‐Dex and BDP‐ELP at 35 °C. Red is ELP‐*b*‐Dex and blue indicates monoblock ELP. c) Cytofluorogram of fluorophores of rhodamine (ELP‐*b*‐Dex) and BODIPY (ELP[M_1_V_3_‐60]) showing colocalization analysis with Pearson's correlation constant *ρ* = 0.814. d) Fluorescence colocalization imaging of co‐assembled ELP‐*b*‐PEG and BDP‐ELP at 35 °C. Red is ELP‐*b*‐PEG and blue is monoblock ELP. e) Cytofluorogram of fluorophores of rhodamine (ELP‐*b*‐PEG) and BODIPY (ELP[M_1_V_3_‐60]) showing colocalization analysis with Pearson's correlation constant *ρ* = 0.879. f) Determination of transition temperatures from DCR measurement of three systems in 8 wt% PEG with operating temperature from 10 to 60 °C, namely 0.25 mg mL^−1^ ELP[M_1_V_3_‐60], 0.25 mg mL^−1^ ELP[M_1_V_3_‐60] + 0.125 mg mL^−1^ ELP‐*b*‐Dex and 0.25 mg mL^−1^ ELP[M_1_V_3_‐60] + 0.125 mg mL^−1^ ELP‐*b*‐PEG. All three systems show a sharp phase transition ≈30 °C. g) DLS size distribution of three systems in 8 wt% PEG above the transition temperature at 35 °C. h) DLS size distribution of two individual ELP‐polymer (0.125 mg mL^−1^ in 8 wt% PEG) at 35 °C and 55 °C, respectively.

Heating the multi‐component complex system to 35 °C above the *T*_cp_ of the monoblock ELP (but below the *T*_cp_ of ELP‐polymer) induced rapid phase separation of BDP‐ELP forming ELP‐rich droplets and their spatial re‐organization at the dextran/PEG interface, as expected (**Figure** **6**a). Nevertheless, it was surprising to see at this temperature (below the *T*_cp_ of ELP‐polymers) a colocalization of monoblock ELP with ELP‐polymers with a clear overlay of rhodamine and BODIPY that are used to label ELP‐polymers and monoblock ELP, respectively (Figure 6b,d). To quantify the colocalization of ELP‐polymer and the monoblock ELP, fluorescence colocalization analysis was carried out based upon a Pearson's correlation constant *ρ* that varies between −1 and 1, where 1 means perfect correlation between two fluorescence intensities and inversely −1 is for perfect anti‐correlation. The *ρ* values measured for ELP‐*b*‐Dex and ELP‐*b*‐PEG were respectively 0.814 and 0.879 (Figure 6c,e; see details in Supporting Information), confirming a good arrangement of colocalization between fluorescence intensities emitted from the monoblock ELP and ELP‐polymer conjugates. These observations indicated that the monoblock ELP coacervates may thus act as metastable nuclei that were spontaneously stabilized by ELP‐polymers acting as surfactants. Surprisingly, these diblock/monoblock co‐assembled colloids exclusively localized at the dextran/PEG interface of the ATPS, which might due to particulates tend to stabilize the aqueous–aqueous interface from lowering the surface tension between dextran and PEG phases.^[^ ^21^ ^]^ Such a strong interface‐enriched redistribution differed from the spatial organization of phase‐separated ELP‐polymer assemblies previously observed (Figure 4), even if the global concentration of ELPs is larger. We could simply explain this phenomena as the size the diblock/monoblock co‐assembled colloids was much larger than that of the diblock coacervate‐core micelles (Figure 6g,h), thus significantly decreasing the overall available surface of the resulting colloids (Figure S12, Supporting Information).

Upon heating to 50 °C, no change in terms of both fluorescence colocalization or partitioning could be observed (Figure S13, Supporting Information), thus confirming a complete co‐assembly mechanism already occurring at 35 °C, a temperature far below the *T*_cp_ of the ELP‐polymer bioconjugates. To further study this mechanism at macromolecular level, we performed dynamic light scattering measurements (DLS, see details in Supporting Information) for three solutions based on 8 wt% PEG (BDP‐ELP alone; ELP‐*b*‐Dex/BDP‐ELP; ELP‐*b*‐PEG/BDP‐ELP) from 10 °C, gradually crossing the *T*_cp_ of the BDP‐ELP and the *T*_cp_ of ELP‐polymers, finally to 60 °C. As shown in Figure 6f, all three system evidenced a clear and unique increase in light scattering starting ≈30 °C, hallmark of the co‐assembly mechanism. Moreover, for all‐three systems, we obtained stable colloidal structures at 35 °C with sizes ≈750 nm (Figure 6g and Figure S15a, Supporting Information). In addition, when similar measurements were conducted on ELP‐polymers in the absence of the monoblock ELP (Figure 6h and Figure S15b, Supporting Information), the formation of nanostructures (sizes ≈230 nm) only occurred above 50 °C, a temperature above *T*_cp_ of ELP‐polymers. We further verified that this co‐assembly mechanism was not due to the crowding environment created by the ATPS. Experiments performed in water (Figures S14 and S15c,d, Supporting Information) evidenced exact same behavior, with an expected small shift in transition temperatures. Furthermore, to demonstrate the reversibility of this dynamic co‐assembly effect, the temperature was cooled down to 10 °C. In both cases of ELP‐*b*‐Dex and ELP‐*b*‐PEG, the co‐assembled constructs from ELP‐polymers and ELP coacervates, readily re‐solubilized and redispersed, leading back to the same original partition into dextran or PEG phases (Figure S16, Supporting Information). During the regulation of temperature for this complex system that includes both the monoblock ELP and an individual ELP‐polymer conjugate, dextran lumen maintained its intact shape, phase separating from the surrounding PEG phase, that is because of the selected ration between dextran and PEG phase can guarantee a two‐phase form in the temperature range from 10 to 50 °C. The ability to dynamically co‐assemble with ELP coacervates in this fashion of molecular engineering of the ELP upon conjugation to particular polymers, is a significant advance in creating a new class of organelle‐mimicking systems capable of sequestering hydrophobic molecules and assemblies within cytomimetic protocells.

Altogether, we believe these findings of spatially localized co‐assembly of the monoblock ELP and ELP‐polymer conjugates will enable a facile and versatile way for interfacial constructing functional organelle mimics.^[^ ^24^ ^]^

## 3 Conclusion

We have presented molecularly engineered bioconjugates via covalent conjugation between an artificial IDP model (ELP) and biocompatible polymers, either dextran or PEG so as to bottom‐up construct temperature‐responsive organelle‐mimics within synthetic cytoplasm containing cell‐like compartments. Combining both ELP‐polymers (ELP‐*b*‐Dex and ELP‐*b*‐PEG) and dextran/PEG paired crowded milieu inside protocells allowed the systematic study of ELP‐polymers’ ability of phase separation and spatial distribution through regulating the temperature. Particularly, the affinity‐based enrichment of ELP‐*b*‐PEG artificial organelles in specific PEG‐rich phase provided a new route to design, assemble and position organelle‐like bodies in a highly selective fashion, like naturally occurring spatially patterned and multilayered intracellular organelles.^[^ ^25^ ^]^ Furthermore we demonstrated that both ELP‐polymer bioconjugates are able to dynamically and reversibly co‐assemble with the monoblock ELP, further stabilizing ELP‐rich coacervates by mediating the temperature above the monoblock ELP transition temperature. Taken together, rational design and synthesis of new building blocks based upon the ELP in this molecular engineering manner is a critical step forward constructing more complex and realistic synthetic organelles that hold promise for furthering our understanding of intracellular organelles featured with high degree of hierarchical complexity in living cells.

## 4 Experimental Section

### Materials

PEG 8 kDa and dextran 500 kDa polymers were purchased from Alfa Aesar. Fluorescein isothiocyanate (FITC)‐dextran 500 kDa conjugate was from Sigma‐Aldrich. The following reagents were purchased from Sigma‐Aldrich and used as they received: sodium chloride (NaCl, VWR, 100%), copper (II) sulfate pentahydrate (CuSO_4_ 5H_2_O, 99%), anhydrous magnesium sulfate (MgSO_4_, 99.5%), N‐hydroxysulfosuccinimide sodium salt (sulfo‐NHS, 98%), 2‐(N‐morpholino) ethanesulfonic acid hydrate (MES, 99.5%), N,N‐diisopropylethylamine (DIPEA, 99%) and mineral oil. Sodium ascorbate (NaAsc, 99%) was obtained from Alfa Aesar. Methoxypolyethylene glycol (mPEG2000) and N‐(3‐Dimethylaminopropyl)‐N′‐ethylcarbodiimide hydrochloride (EDC, 98%) were purchased from TCI. Rhodamine cadaverine was purchased from VWR. Cuprisorb was purchased from Seachem. 5‐(6)‐carboxy‐x‐rhodamine *N*‐succinimidyl ester (C_37_H_33_N_3_O_7_, Chemodex, 95%) was bought from Adipogen. Phosphate buffer solution (10×, pH 7.4) was purchased from Euromedex. Dialysis was conducted using a Spectra/Por6 dialysis membrane. The following solvent was used without further purification: acetonitrile (ACN, VWR chemicals, HPLC grade, 99.9%), diethyl ether (Et_2_O, VWR chemicals, 97%) and dimethyl sulfoxide (DMSO, Sigma‐Aldrich, 99.9%). Water with a resistivity of 18.2 MΩ cm^−1^ is prepared using a Millipore Milli‐Q system.

### Construction of the Expression Vector

The sequence coding for MW[VPGVGVPGMG(VPGVG)_2_]_10_ was obtained by using a variation of the recursive directional ligation,^[^ ^26^ ^]^ adapting the procedure described previously.^[^ ^18a,b^ ^]^

### Bioproduction, Isolation, Purification, and N‐Terminal Post‐Modification of Recombinant ELP[M_1_V_3_‐40]

ELP[M_1_V_3_‐40] was produced by recombinant DNA and protein engineering techniques in *E. coli* and isolated using previously reported procedures.^[^ ^18a,b^ ^]^ *N*‐terminal was modified by 4‐pentynoic acid succinimidyl ester to obtain alkyne‐ELP[M_1_V_3_‐40] as previously reported by our group elsewhere.^[^ ^18c^ ^]^

### Fabrication of Glass Capillary Devices

The microfluidic device used to generate paired water‐in‐oil emulsions here were assembled from cylindrical and square glass capillaries. To emulsify two aqueous phases mixed with dextran/ELP‐polymer conjugates and PEG together into an oil phase, theta‐shape capillary (World precision instruments, TST150‐6) of 1.5 mm outer diameter was tapered using micropipette puller (Sutter instrument, P‐97) followed by polishing with sand papers. This tapered theta‐shape capillary was treated with chlorotrimethylsilane (Sigma‐Aldrich) rendering its surface into hydrophobic, and then inserted into a square capillary (VitroCom, 3015C2) with the inner dimension of 1.58 mm. To collect the paired microdroplets formed and delivered by the continuous phase, the open end of a circular capillary of 1.5 mm outer diameter (World precision instruments, 1B150‐6) was inserted into square capillary in opposite direction. Lastly, for the microfluidic device, dispensing needles used as inlets of fluids were connected at the junctions between capillaries by using a transparent 5 min Epoxy (Devcon). The device was connected to high‐precision syringe pumps (Chemyx, Fusion 100) via silicone tubing (VWR, 1/3 mm inner/outer diameter) to ensure reproducible, stable flows. In the scenario of preparing water‐in‐oil single emulsions where ELP‐*b*‐PEG/ELP‐*b*‐Dex and PEG solution were mixed as an aqueous core, two round capillaries of 0.58 mm in inner dimension (World precision instruments, 1B100‐4) were tapered and the tips were polished to 60 and 150 µm using as injection and collection channel, respectively. The capillary with smaller tip was modified by chlorotrimethylsilane (Sigma‐Aldrich) into hydrophobic to pump the aqueous solution. These two capillary tubes were inserted oppositely into a square capillary (VitroCom, 2956C1) with the inner dimension of 1.05 mm. Similarly, needles were placed at each junction and sealed using the 5 min Epoxy.

### Water‐in‐Oil Microdroplet Formation

To create paired water‐in‐oil emulsion droplets, two aqueous phases—8 wt% dextran phase contains FITC‐dextran and ELP‐*b*‐PEG or ELP‐*b*‐Dex, and 16 wt% PEG solution were flowed in two separate channels of the theta‐shape microcapillary as dispersed phase. At the tip of this injection capillary where the two polymeric solutions meet, an organic, continuous phase comprised of 75/20/5 vol% TEGOSOFT DEC (Evonik Industries, Germany)/mineral oil/ABIL EM 90 (Evonik Industries, Germany) emulsified the aqueous phase forming monodispersed water‐in‐oil droplets. Flow rates of two aqueous fluids and continuous phase were 100 µL h^−1^ and 12 mL h^−1^, respectively, which are tuned to ensure droplet formation in the dripping regime. When BDP‐ELP[M_1_V_3_‐60] was included, two aqueous phases were 8 wt% dextran + 0.5 mg mL^−1^ BDP‐ELP[M_1_V_3_‐60] and 16 wt% PEG + 0.25 mg mL^−1^ ELP‐polymer conjugate, respectively, which were pumped into the microfluidic device. As to generate water‐in‐oil single emulsions for visualizing and investigating position of phase‐separated ELP‐polymer conjugates in PEG phase only, a mixture of an individual ELP‐polymer and PEG solution was prepared as dispersed fluid. The same organic phase was pumped into microfluidic device to pinch off the water phase into monodispersed water‐in‐oil droplets. The flow rates of the dispersed and organic continuous fluids were 500 and 5000 µL h^−1^ that are tuned to ensure droplet formation in the dripping mode.

### Heating/Cooling and Imaging

Paired water‐in‐oil emulsions were collected on a glass slide with a single cavity (Sigma‐Aldrich) and subsequently were sealed with a cover slip. The emulsion samples were heated/cooled using a precise Peltier temperature‐controlled microscope stage (Linkam, PE100) equipped with a Linkam PE95 digital temperature control unit. A rate of 10 °C min^−1^ for increasing and decreasing temperature was applied to all samples. Images were acquired by a confocal laser scanning microscopy (Leica, SP5 AOBS) through an HCX PL APO 10× dry objective and an HCX L APO 40× water immersion objective. To assess localization of ELP‐*b*‐PEG/ELP‐*b*‐Dex and ELP[M_1_V_3_‐60] and monitor their coacervation and spatial distribution, they were labeled with spectrally different fluorophores. An argon laser (488 nm), diode laser (561 nm) and He‐Ne (633 nm) ion laser were used to excite FITC, rhodamine and BODIPY, respectively. To avoid an artifact of visualizing fluorescent conjugates and BODIPY‐labeled ELP[M_1_V_3_‐60], sequential imaging mode was used to reduce fluorescence crosstalk among various fluorophores.

### FRAP

FRAP was performed using the FRAP‐Wizard of the Leica LAS‐AF microscope software which allowed to control and tune the scanning conditions: prebleach, photobleach, and postbleach phases. Rhodamine‐labeled ELP‐polymer conjugates were excited and bleached with the 561 nm laser line. ROIs were defined on the dextran/PEG interface with a diameter of 10 µm. FRAP acquisition was started with ten images scan at low laser power. Then, the dye was bleached locally inside the ROIs at 100% laser power using a scan of 10 frames. Finally, fluorescence recovery was monitored by the acquisition of a series of 150 images at the same low laser power as the prebleach phase.

### Statistical Analysis

For determination of partitioning fraction between dextran‐rich and PEG‐rich phase of either ELP‐Dex or ELP‐PEG, results were displayed as mean ± standard deviations. The sample sizes (*n*) were provided in the figure legends. Concerning the measurements of *T*_cp_, all data of derived count rate were normalized from 0 to 1 by using the software Origin (OriginLab Corp).

## Conflict of Interest

The authors declare no conflict of interest.

## Supporting information

Supporting Information

## Data Availability

Research data are not shared.
