# Supplementary material for: Spatiotemporal Dynamic Assembly/Disassembly of Organelle‐Mimics Based on Intrinsically Disordered Protein‐Polymer Conjugates
Source: Adv Sci (Weinh). 2021 Nov 1;8(24):2102508. doi: 10.1002/advs.202102508 (PMC8693077; doi:10.1002/advs.202102508)
Supplement: Supplementary file 1 — Supporting Information [file ADVS-8-2102508-s001.pdf]

Copyright WILEY-VCH Verlag GmbH & Co. KGaA, 69469 Weinheim, Germany, 2018.

# Supporting Information

## Spatiotemporal Dynamic Assembly/Disassembly of Organelle-Mimics Based on Intrinsically Disordered Protein-Polymer Conjugates

*Hang Zhao, Emmanuel Ibarboure, Vusala Ibrahimova, Ye Xiao, Elisabeth Garanger and Sébastien Lecommandoux**

### Synthesis and Purification of Fluorescently Labelled ELP[$M_1V_3$-40]

DIPEA (1 equiv.) was added to a solution of ELP[$M_1V_3$-40] (10 mg) in anhydrous DMSO (1 mL). After stirring the solution rhodamine-NHS ester (2 equiv. 5-(6)-carboxy-x-rhodamine *N*-succinimidyl ester) was added and the reaction was left under the stirring for 24 hours at room temperature under $N_2$ in the dark. Then the mixture was precipitated into the diethyl ether and centrifuged. Precipitate was dissolved in cold water and purified by inverse transition cycling (ITC). The final pale pink product Rh-ELP[$M_1V_3$-40] was obtained by lyophilization. (7 mg, 70% product yield). In order to purify fluorescent ELP product, precipitate was dissolved in 2 mL cold water and a few drops of 1.5 M NaCl solution was added into the solution. Tube was placed in heating bath (40 °C) to fluctuate products and enhance the separation from free dye molecules. Solution was centrifuged 30 minutes at 38 °C (3800 rpm speed) and supernatant was discarded. The dark pink pellet was dissolved in 3 mL cold water and few drops of 1.5 M NaCl solution was added into the supernatant and placed into the heating bath (40 °C) and centrifuged 30 minutes at 38 °C (3800 rpm speed). This process was repeated until the clear supernatant was observed. Finally, the supernatant was discarded and the pellet was dissolved in cold Milli-Q water. To remove the excess salt, the solution was washed tree times with cold water by ultrafiltration technique. The final solution was lyophilized to obtain pure pale pink Rh-ELP[$M_1V_3$-40].

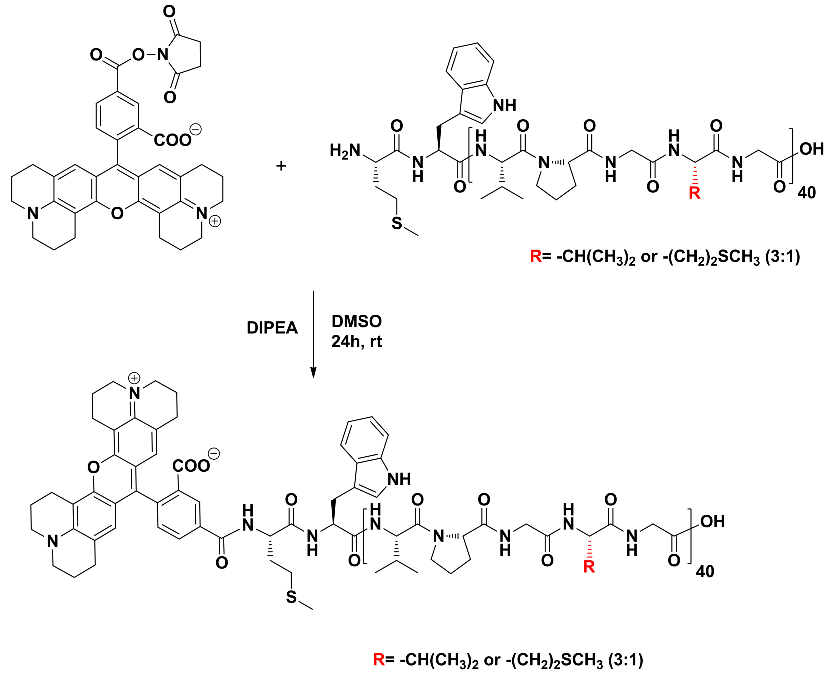


**Scheme S1.** Synthesis of rhodamine-labelled ELP[$M_1V_3$-40], noted Rh-ELP[$M_1V_3$-40].

### Synthesis of α-Methoxy ω-Azido Poly(ethylene glycol) (mPEG-$N_3$)

The chain end of methoxypolyethylene glycol (mPEG) (MW 2 kDa) was converted into an azide group by following literature procedures.[1]

### Synthesis of ELP-*b*-PEG Conjugate

mPEG-$N_3$ (12.3 mg, 5.8 μM), copper sulfate (4.4 mg, 17.6 μM) and sodium ascorbate (7 mg, 5.2 μM) were added to a solution of *alkyne*-ELP[$M_1V_3$-40] (50 mg, 2.9 μM) in anhydrous DMSO (5 mL) under argon atmosphere. The reaction was stirred at room temperature for 3 days, after which the mixture was diluted with cold water (12 mL). Cuprisorb (90 mg) was added to the resulted solution and it was then incubated at room temperature with shaking, for overnight to remove the copper. Cuprisorb was removed by centrifuge and the supernatant was purified by dialysis (MWCO 15 kDa) against ultrapure water for 5 days (changing water 3 times per day), followed by lyophilization to obtain the final product as a white powder (53 mg, 91% yield).

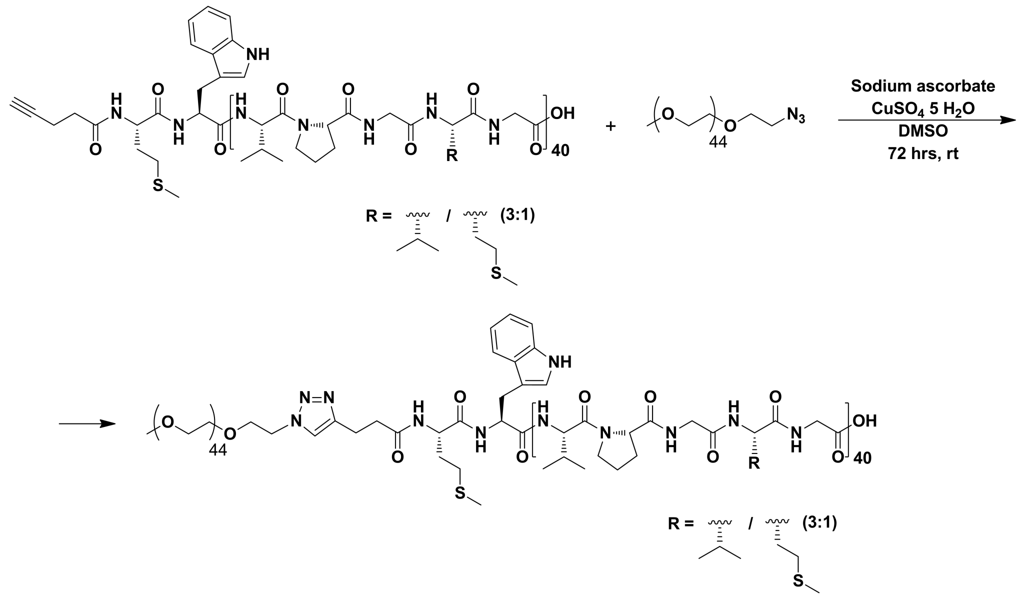


**Scheme S2.** Copper-catalyzed azide-alkyne cycloaddition reaction between the azide-functionalized mPEG-$N_3$ and *alkyne*-ELP[$M_1V_3$-40].

$^1$H NMR (400 MHz, $D_2O$): δ 7.78 (s, triazole *H*), 7.60-7.09 (br, indole *H* Trp), 4.55 (m, C$H_\alpha$ Met), 4.44 (m, C$H_\alpha$ Val, Pro), 4.17 (d, C$H_\alpha$ $Val_{Xaa}$), 4.04-3.86 (br m, C$H_{2\alpha}$ Gly, C$H_{2\delta}$ Pro), 3.77-3.60 (m, C$H_{2'\delta}$ Pro, mPEG -OC$H_2$C$H_2$O-), 3.39 (mPEG -OC$H_3$), 2.68-2.48 (br m, C$H_{2\gamma}$ Met), 2.40-2.25 (m, C$H_{2\beta}$ Pro), 2.20-1.87 (m, C$H_{2\beta}$ Met, C$H_{2'\beta}$ Pro C$H_{2\gamma}$ Pro, C$H_\beta$ Val, C$H_{3\varepsilon}$ Met), 1.04-0.86 (m, C$H_{3\gamma}$ Val). Size exclusion chromatography (SEC) (in buffer 0.1 M $NaNO_3$, 0.01 M $Na_2HPO_4$, 0.02 M $NaN_3$) MW 19.2 kDa, Đ = 1.04, MALDI-MS $[M+H]^+$ 19126.46 Da.

**Synthesis and Purification of Fluorescently Labelled ELP-*b*-PEG and ELP-*b*-Dex**

ELP-*b*-PEG or ELP-*b*-Dex (1 equiv.) was dissolved in MES buffer (pH 5) at 10 mg mL$^{-1}$ concentration. EDC (20 equiv.) and sulfo-NHS (10 equiv.) were added to a solution and left under stirring for 1h at ambient temperature. The solution was adjusted to pH ~7 with PBS buffer. Rhodamine cadaverine (10 equiv.) was immediately added into the reaction mixture and left under the stirring for 24 hours at room temperature in dark. The excess free dye molecules, ELP and the reagents were eliminated by washing the solution 5 times with Milli-Q water in the centrifugal filter (Amicon Ultra-15, Ultracel-10K). The final concentrated

solution was lyophilized to obtain pure conjugates (18 mg, 90% product yield for ELP-*b*-PEG and 15 mg, 75% product yield for ELP-*b*-Dex). The labelling and purification were confirmed by SDS-PAGE analysis method.

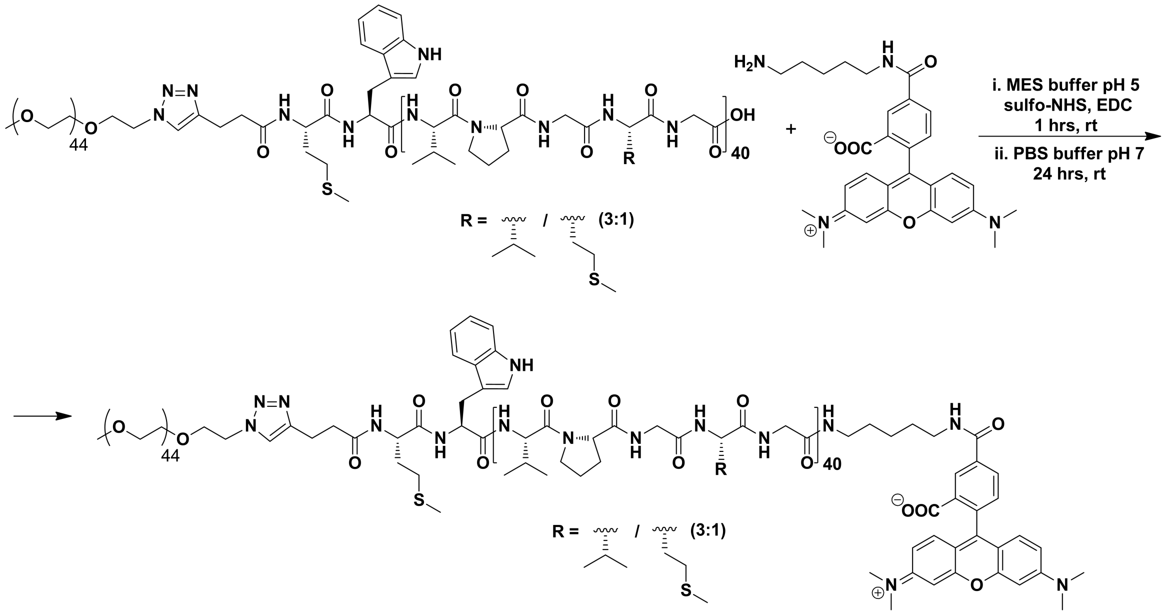


**Scheme S3.** *C*-terminus labelling of ELP-*b*-PEG by rhodamine cadaverine *via* carbamide coupling reaction in aqueous environment.

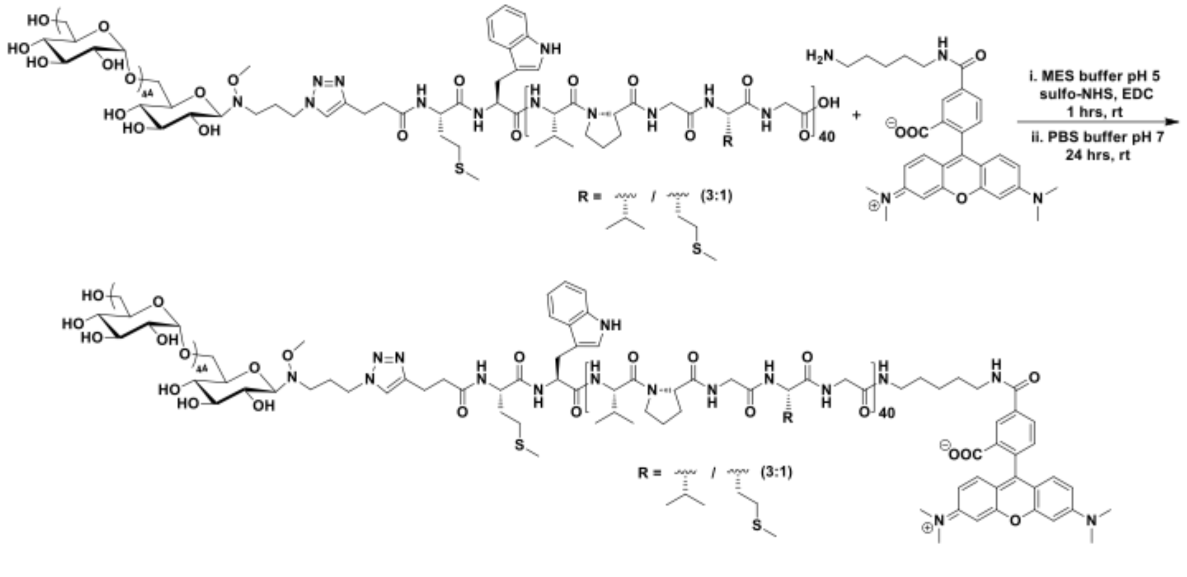


**Scheme S4.** *C*-terminus labelling of ELP-*b*-Dex by rhodamine cadaverine *via* carbamide coupling reaction in aqueous environment.

**Nuclear Magnetic Resonance (NMR)**

$^1$H NMR 400 MHz spectra have been taken with a Bruker Avance I (Liquid-state 400 MHz NMR spectrometer with 5 mm BBFO probe). Deuterated chloroform ($CDCl_3$, Euriso-top, 99.8%), deuterated dimethylsulfoxyde (d-DMSO, Euriso-top, 99.8%) and deuterated water ($D_2O$, Euriso-top, 99.8%) were used as solvent and reference for the lock.

**Size Exclusion Chromatography (SEC)**

For ELP-polymer conjugates: SEC analysis was performed on a SEC-MALS system with refractive index detector (WYATT Technology Optilab rEX and HELEOS-II) using an aqueous buffer (0.1 M $NaNO_3$, 0.01 M $Na_2HPO_4$, 0.02 M $NaN_3$) with a flow rate of 0.6 mL min$^{-1}$ at 22 °C. The specific 88 refractive index increment (dn/dc) of conjugate was measured by means of a differential refractometer (Wyatt Optilab rEX) operating at a wavelength of 658 nm at 26 °C. A single concentration of each conjugate was used to determine the dn/dc coefficient through the calculation module implemented in the Astra 7.1 software. The measured dn/dc values of conjugate were applied for the calculation of weight average molecular weight (MW).

For ELP[$M_1V_3$-40]: SEC measurement in Water/ACN was performed on an Ultimate 3000 system from Thermoscientific equipped with diode array detector DAD. The system also includes a multi-angles light scattering (18 angle) detector MALS and differential refractive index detector dRI from Wyatt technology. Polymers were separated on Tosoh G3000+G4000 (7.8*300) columns (exclusion limits of G4000 from 2 kDa to 300 kDa and G3000 40kDa) at a flowrate of 0.6 mL min$^{-1}$, 35 bar pressure. Columns temperature was held at 26 °C. Polyethylene glycol was used as the standard. Water/ACN (65:35 vol% + ammonium acetate 0.2 M and acetic acid 0.3 M) mixture were used as the eluent.

**Mass Spectrometry Analysis (MS)**

MALDI-MS spectra were performed by the CESAMO (Bordeaux, France) on an Autoflex maX TOF mass spectrometer (Bruker Daltonics, Bremen, Germany) equipped with a frequency tripled Nd:YAG laser emitting at 355 nm. Spectra were recorded in the linear positive-ion mode with an accelerating voltage of 19 kV. Samples were dissolved in water at 4 mg $mL^{-1}$. The SA matrix (sinapinic acid) solution was prepared by dissolving 10 mg in 1 mL of acetonitrile/0.1% aqueous TFA 50/50. The solutions were combined in a 10:10 volume ratio of matrix to sample. One to two microliters of the obtained solution were deposited onto the sample target and vacuum dried.

**Dynamic Light Scattering (DLS)**

To determine the transition temperature of ELP-polymer conjugates and to assess the co-assembly of individual ELP-polymer conjugate and ELP[$M_1V_3$-60] at macromolecular level, dynamic light scattering (DLS) measurements were performed on NanoZS instrument (Malvern, U.K.) at a 90° angle at a constant position in the cuvette (constant scattering volume). The derived count rate (DCR) was defined as the mean scattered intensity normalized by the attenuation factor. The DCR was plotted against temperature and the $T_{cp}$ is defined as the temperature corresponding to the point where the DCR starts increasing on the plot.

**Electrophoresis (SDS-PAGE) Analysis**

Final purity of the fluorescently tagged ELP and ELP-polymer conjugates were assessed by SDS-PAGE electrophoresis. 15 µL of Precision Plus Protein Standards (size marker) and 20 µL of solution from the sample were loaded on the gel trays (BIO-RAD, 4-20% Mini-PROTEAN® TGX™ stain-free gel). Tris-Glycine-SDS Buffer (TGS 1x) was used as the loading sample buffer.

**Determination of Colocalization Coefficient**

To assess the colocalization of each ELP-polymer conjugate with ELP[$M_1V_3$-60], confocal microscopy images were analyzed using ImageJ/Fiji software.[2] The corrected images were analyzed using the JaCoP plugin in ImageJ/Fiji to obtain Pearson's correlation coefficient.

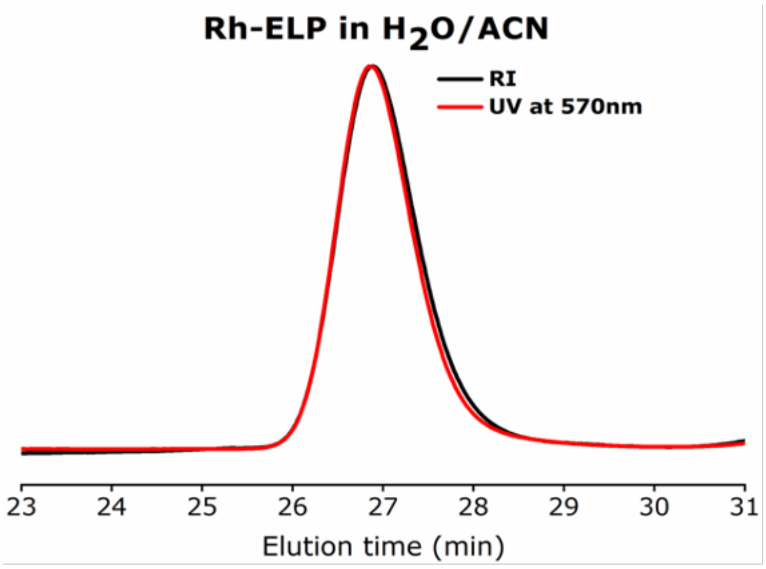


**Figure S1.** Size exclusion chromatogram of Rh-ELP[$M_1V_3$-40] in Water/ACN.

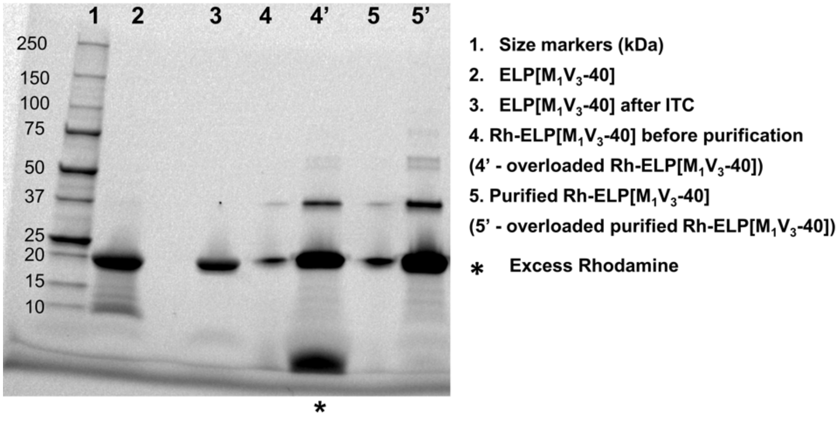


**Figure S2.** SDS-PAGE analysis of Rh-ELP[$M_1V_3$-40] (unstained gel).

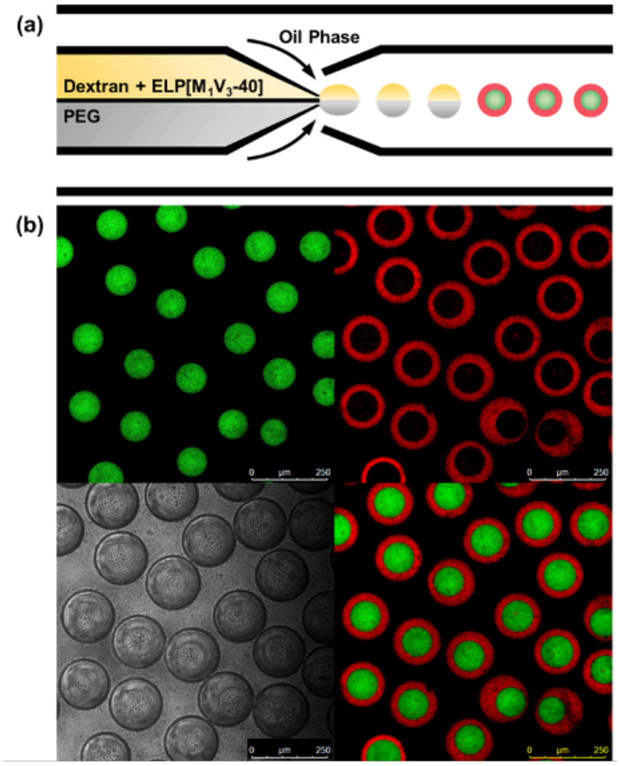


**Figure S3.** Evaluation of the preferential localization of monoblock ELP[$M_1V_3$-40] in the cytomimetic protocellular compartments. (a) Scheme of generation of single emulsion using a microfluidic device. Rhodamine-labelled ELP[$M_1V_3$-40] was pumped into the microfluidic device together with dextran phase in the same channel. At the orifice of the *θ*-shape capillary, two aqueous phases met and were pinched-off by the oil solution, forming the paired emulsions. (b) Imaging the paired emulsions by confocal microscopy at 10 °C confirms that the specific accumulation of ELP[$M_1V_3$-40] into the PEG-rich phase, which is similar to what we have reported elsewhere regarding on the ELP with longer length.[3]

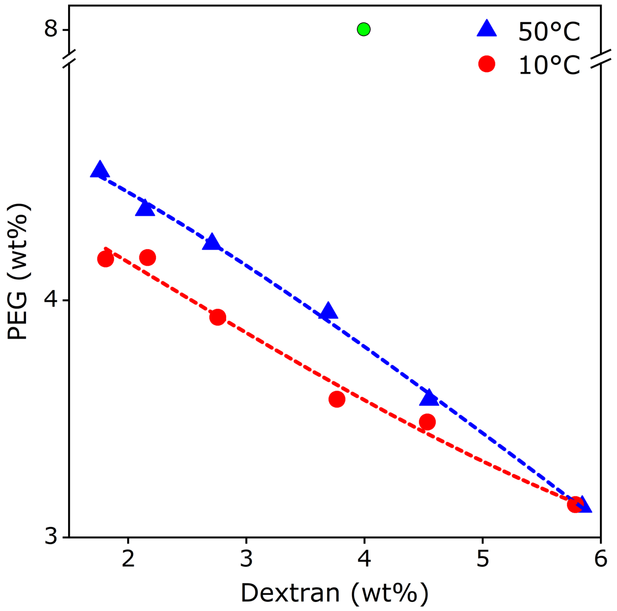


**Figure S4.** Experimentally determined phase diagram for dextran 500 kDa and PEG 8 kDa at 10 °C (red circle) and 50 °C (blue triangle). Lines are to guide the eye. The typical concentrations of dextran and PEG used in experiments were 4 wt% and 8 wt% (indicated by the green circle), respectively. The composition ratio locates above both curves for 10 and 50 °C, indicating the dextran/PEG system remains phase-separated condition between 10 and 50 °C.

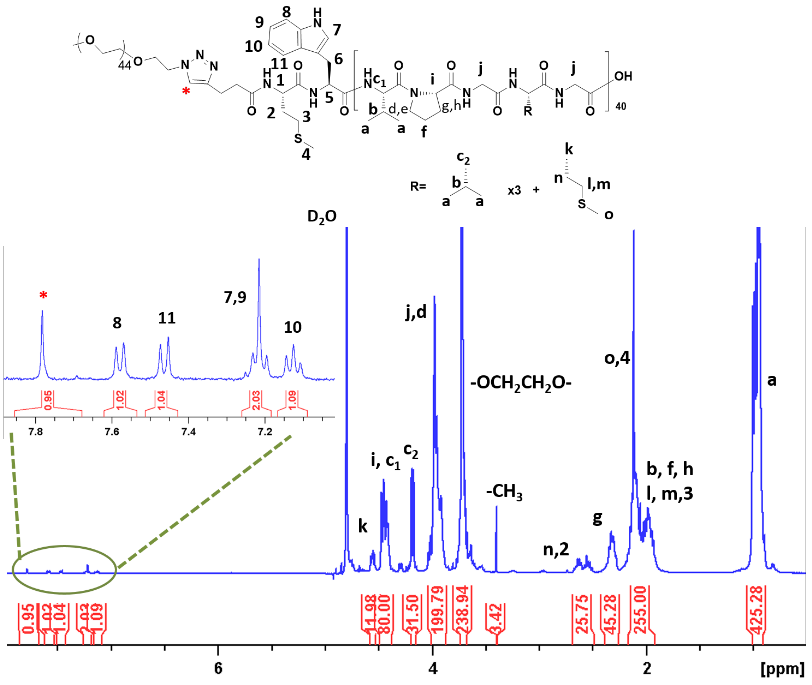


**Figure S5.** $^1$H NMR spectrum of ELP-*b*-PEG in $D_2O$.

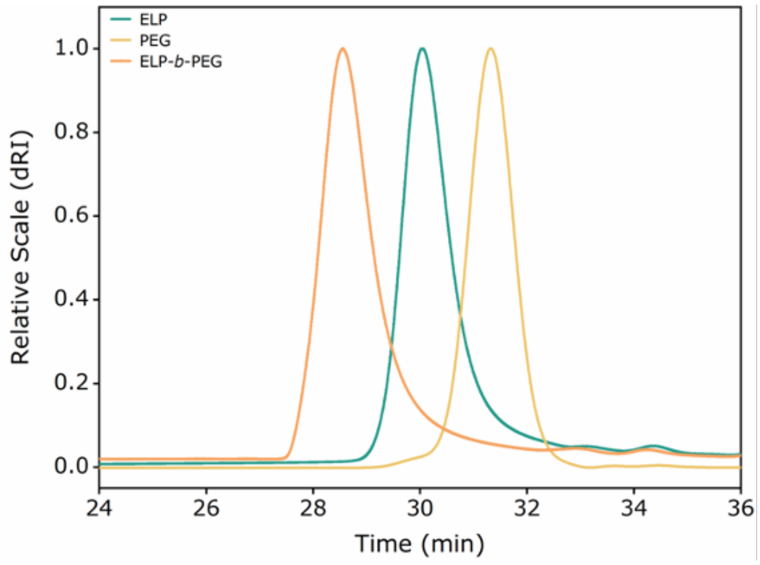


**Figure S6.** Size exclusion chromatograms (RI detection) of PEG, ELP[$M_1V_3$-40] and ELP-*b*-PEG conjugate in aqueous buffer (0.1 M $NaNO_3$, 0.01 M $Na_2HPO_4$, 0.02 M $NaN_3$).

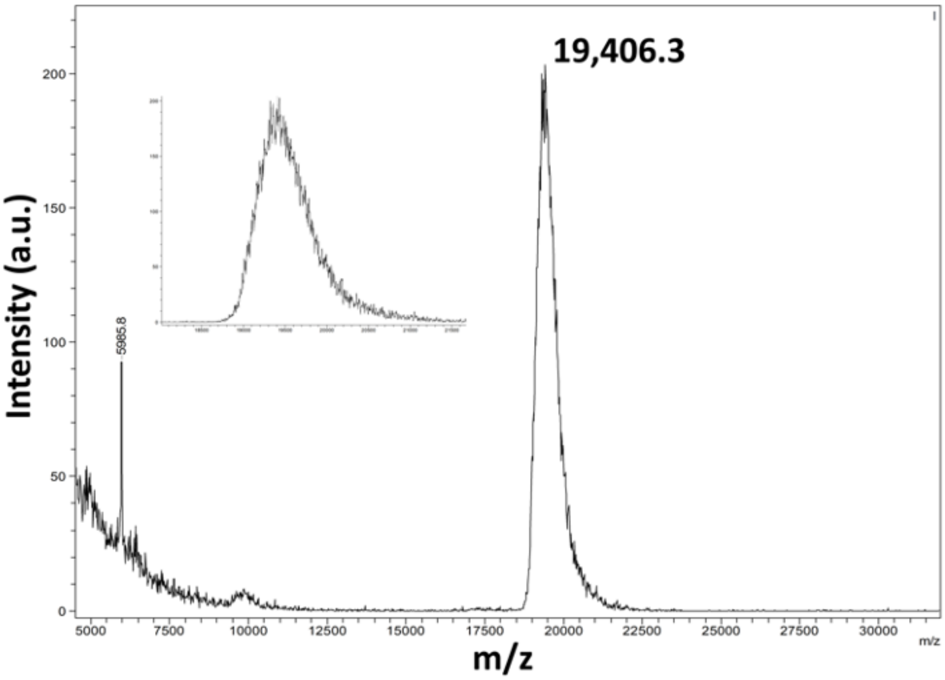


**Figure S7.** MALDI mass spectrum of ELP-*b*-PEG.

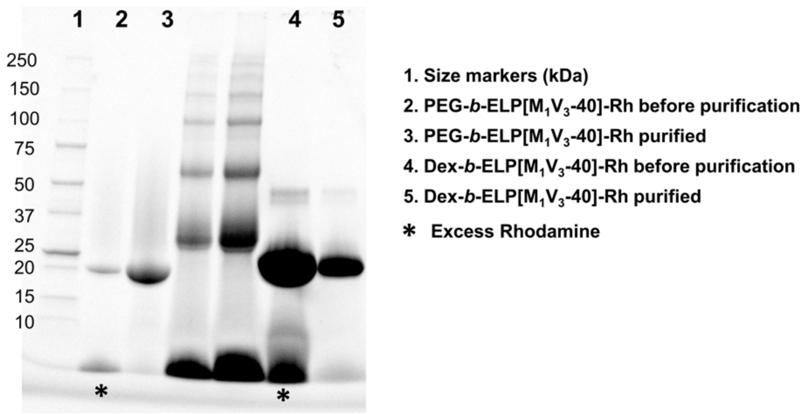


**Figure S8.** SDS-PAGE analysis of the fluorescently labelled conjugates (stain-free gel).

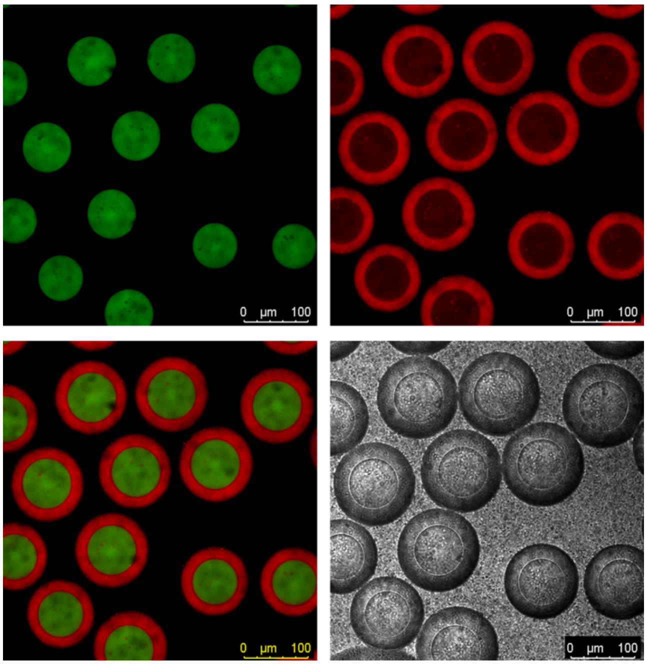


**Figure S9.** To assess if the partitioning fraction of ELP-*b*-Dex between dextran and PEG phases is influenced by associated dissolving solution of the ELP. ELP-*b*-Dex was mixed with 16 wt% PEG phase and was injected into microfluidic device through one channel and met 8 wt% dextran solution at the tip of the injection capillary. The paired emulsions were imaged by confocal microscopy at 10 °C, in which ELP-*b*-Dex remains the similar partitioning ratio between dextran and PEG solutions as observed in the other scenario of mixing ELP-*b*-Dex with dextran phase.

In order to estimate the density difference between ELP-*b*-Dex organelles and PEG-rich phase, we produced microdroplets *via* microfluidics, in which only the individual ELP-polymer conjugate and PEG solution were encapsulated (see details in the Experimental Section) that was followed by increasing the temperature above the ELP-polymers' $T_{cp}$. At 50 °C, both ELP-polymer conjugates exhibited phase-separated behavior forming organelle-like microcompartments. Importantly, ELP-*b*-Dex assemblies were mostly found at the bottom position of hosting microdroplets, indicating the occurrence of sedimentation for ELP-*b*-Dex constructs in the PEG phase. In contrast, the spatially homogenous arrangement of ELP-*b*-PEG aggregates within cell-like droplets was confirmed by the 3D reconstruction from confocal images (Figure S10). In both instances, cooling microdroplets below the $T_{cp}$ dynamically solubilized ELP-polymer phase-separated granules due to ELP chain rehydration, demonstrating the reversible feature of the ELP-polymer organelle-like compartments (Figure S10). Taken together, the results indicated that ELP-*b*-Dex synthetic organelles appeared in both PEG-rich phase and dextran lumen at 50 °C. Organelle assemblies from PEG-rich region dropped to the bottom and were driven to the interface of dextran/PEG phases together with organelle-mimics formed in the dextran lumen to lower the surface tension generates by 4 wt% dextran and 8 wt% PEG.

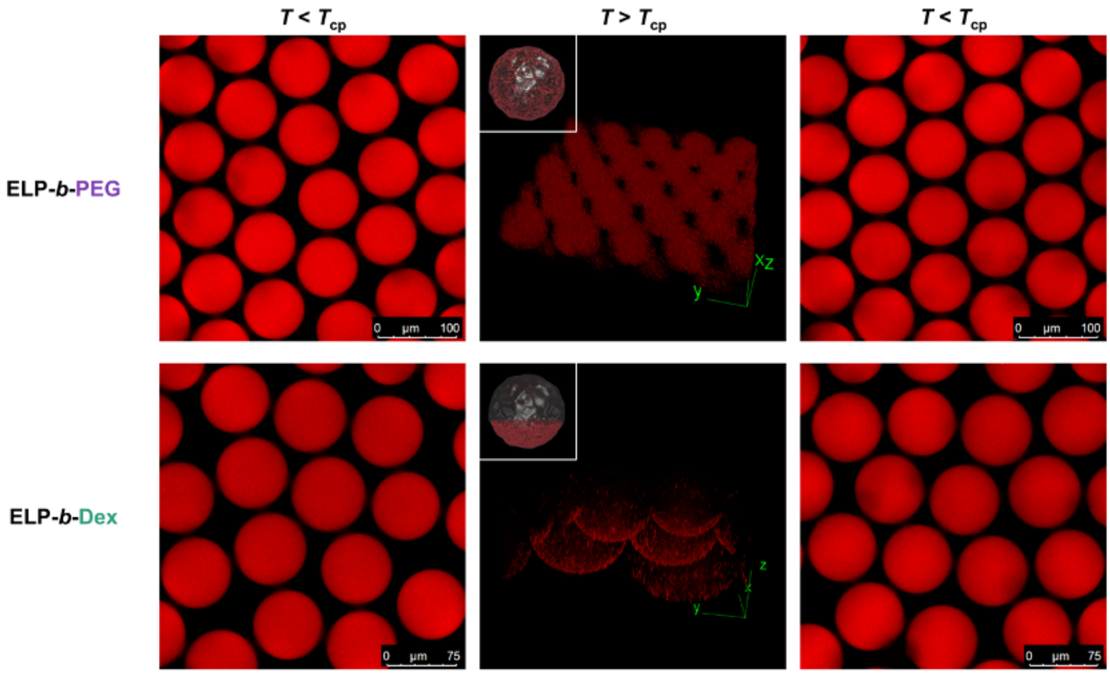


**Figure S10.** Confocal microscopy images of water-in-oil emulsions, in which ELP-polymers and PEG solution were used as dispersed aqueous phase, and 3D reconstruction of droplets demonstrate the distinct spatial positions of two ELP-polymer conjugates in response to temperature. Top panel (ELP-*b*-PEG): Below the transition temperature, fluorescence signal emitted from the ELP-*b*-PEG uniformly distributed within the droplets. When temperature was above $T_{cp}$, as shown in the 3D reconstruction of z-stack images of the droplets, the ELP-*b*-PEG started to phase separate and remained homogenously positioned inside droplets. When the droplets were cooled down below the transition temperature, the formed coacervate-core micelles were hydrated turning back to their original chain-like structures; Bottom panel (ELP-*b*-Dex): When temperature was below the transition temperature, ELP-*b*-Dex molecules were uniform spatially within emulsions; whereas above $T_{cp}$, after the formation of coacervate-core micelles, they sedimented toward the bottom of the droplets. Inset is schematic illustration of distribution of the as-formed coacervate-core micelles within the microdroplets.

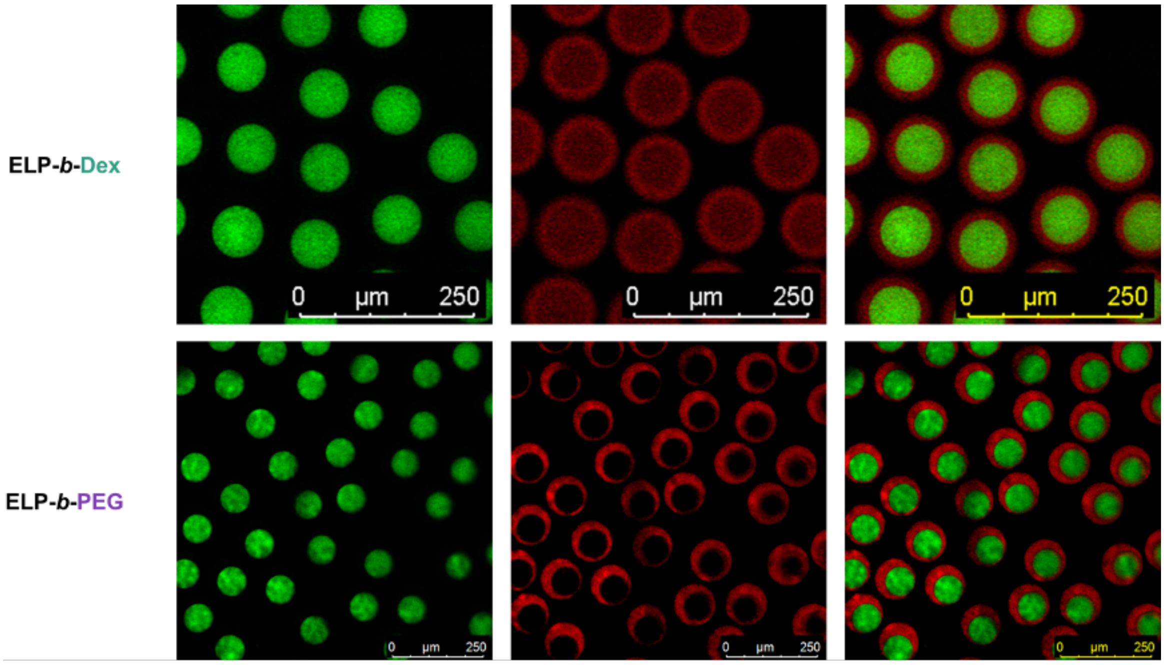


**Figure S11.** Thermally induced reversibility of ELP-polymer conjugates. When temperature is at 10 °C (below $T_{cp}$), ELP-*b*-Dex (top panel) organelle-like bodies disassembled and partitioned to both dextran and PEG phases, while ELP-*b*-PEG phase-separated architectures disassembled as well but enriched remarkably in the PEG-rich phase.

We observed the difference in spatial positioning of two kinds of colloid structures in the ATPS droplets where co-assembled colloids specifically localized at dextran/PEG interface, but ELP-polymer colloids positioned at the interface and in the PEG-rich phase. A simplified model was created to explain this localization difference, considering the concentration of diblock/monoblock ELPs, the size of the colloids formed and the surface of the ATPS interface to be covered (Figure S12).

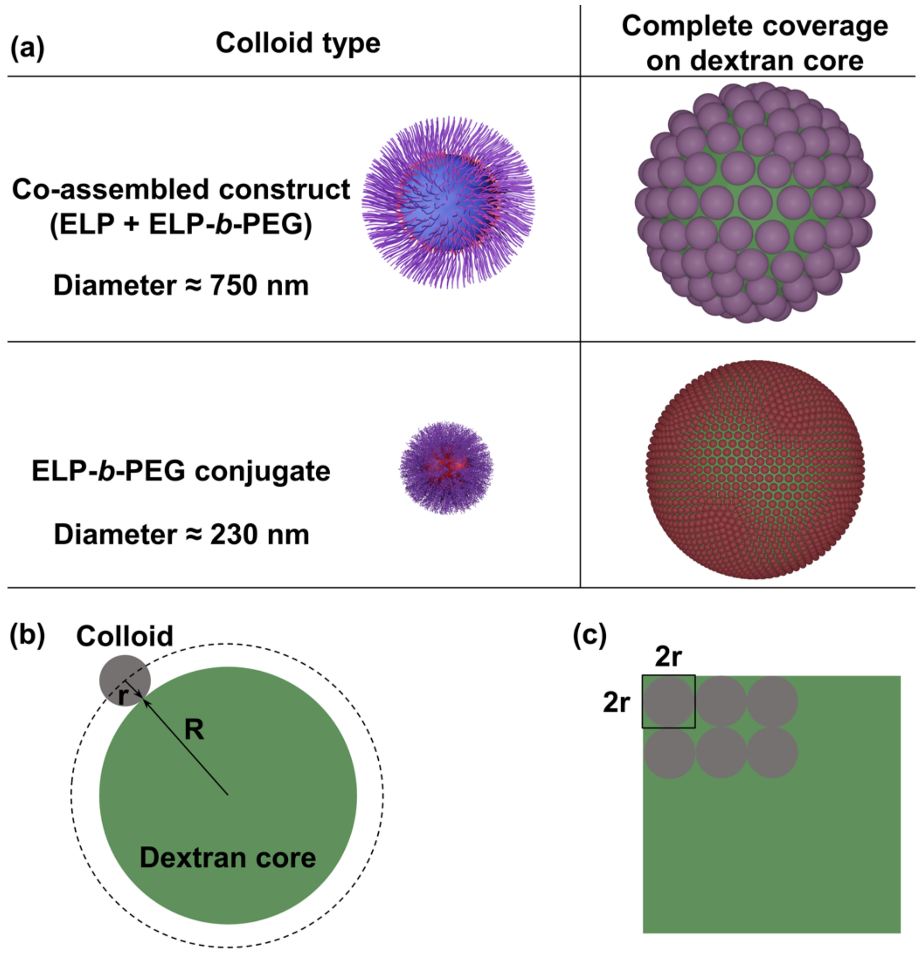


**Figure S12.** Simple schematic model established to explain that co-assembled colloids from the monoblock ELP and ELP-polymer conjugates are only localized at the dextran/PEG interface, whereas phase-separated ELP-polymer coacervate-core micelles are distributed both in the PEG-rich phase and at the interface of dextran/PEG system. (a) Schemes of colloid structures of co-assembled assemblies that are larger in size than that of ELP-polymer coacervate-core micelles (ELP-*b*-PEG is used as an example). Schematic illustrations of completely pack the dextran core by either co-assembled colloids or ELP-polymer assemblies.

(b) Scheme of full coverage of the dextran core with spherical colloids considered as colloid structures are arranged at the spherical surface whose radius is r + R. (c) Scheme of simplified model showing the surface area of the dextran core occupied by each colloid projected from 3D to 2D is a square area $4r^2$.

One can first estimate the maximum numbers of colloidal assemblies (radius r = 375 nm or 115 nm) that are needed to completely cover the dextran interface (radius determined as R = 35 $\mu$m). Assuming the contact between colloids and the dextran core is point contact, to fully cover the surface of the dextran core, we can consider that colloids are aligned closely on a surface whose radius is R + r (Figure S12b). In addition, the area occupied by an individual colloid on the new surface is simplified to a square whose area is $4r^2$ (Figure S12c). Hence, we can obtain the following equation to estimate the maximum number of colloids necessary to stabilize the dextran interface:

$$N_{max} = \frac{4\pi(R+r)^2}{(2r)^2}$$

This gives us $N_{max}$ (ELP conjugate) = 292765 and $N_{max}$ (ELP co-assembled colloid) = 27942. As such, even if the concentration of ELPs is larger in the second case, the number of particles is about 10.4 times smaller, consistent with the formation of larger colloids. This will correspond to *Ratio1*.

As we do not know the exact density of the different coacervates, one can just estimate a *Ratio2* that corresponds to:

$$Ratio2 = \frac{\text{Number of core-shell coacervates from ELP conjugate}}{\text{Number of ELP co-assembled colloid}}$$

Considering the given concentrations in the microdroplets (ELP-polymer: 0.125 mg $mL^{-1}$; BDP-ELP: 0.25 mg $mL^{-1}$ + ELP-polymer: 0.125 mg $mL^{-1}$)

To quantify the number of colloids that are formed in microdroplets, we use:

$$N = \frac{M_{total}}{M_{colloid}}$$

where $M_{total}$ is the total mass of the building block of colloids that are either ELP-polymer alone or ELP-polymer and BDP-ELP; $M_{colloid}$ is the mass of an individual colloid that is either one phase-separated ELP-polymer conjugate or a co-assembled colloid. In addition, $M_{colloid} = d \times V$, where d is the density of a single colloidal structure and V is the volume of the individual colloidal assembly that equals to $\frac{4}{3}\pi r^3$ in which r is the hydrodynamic radius of as-formed colloids. We assume the density for co-assembled colloids and phase-separated ELP-polymer colloids shares the same value in order to simplify the equation.

Therefore, the equation of *Ratio2* can be re-written to

$$Ratio2 = \frac{\frac{M_{total[ELP\text{-}polymer]}}{d \times V_{ELP\text{-}polymer}}}{\frac{M_{total[BDP\text{-}ELP+ELP\text{-}polymer]}}{d \times V_{BDP\text{-}ELP+ELP\text{-}polymer}}} = \frac{M_{total[ELP\text{-}polymer]}}{M_{total[BDP\text{-}ELP+ELP\text{-}polymer]}} \times \frac{V_{BDP\text{-}ELP+ELP\text{-}polymer}}{V_{ELP\text{-}polymer}}$$

Given the total concentration of either ELP-polymer (0.125 mg mL$^{-1}$) or BDP-ELP (0.25 mg mL$^{-1}$)/ELP-polymer (0.125 mg mL$^{-1}$), we can obtain that $\frac{M_{total[ELP\text{-}polymer]}}{M_{total[BDP\text{-}ELP+ELP\text{-}polymer]}} = \frac{1}{3}$.

The volume of each type of colloid was calculated from the hydrodynamic diameters co-assembled colloid and phase-separated ELP-polymer colloid, respectively 750 nm and 230 nm as measured by DLS (Figure 6g and 6h), leading to the result of $\frac{V_{BDP\text{-}ELP+ELP\text{-}polymer}}{V_{ELP\text{-}polymer}} = 33.7$.

Thus,

$$Ratio2 = \frac{1}{3} \times 33.7 = 11.23$$

Taken together, the value of *Ratio2* is larger than that of *Ratio1* validating that ELP-polymer core-shell coacervates remain in excess after completely cover the surface of the dextran droplet.

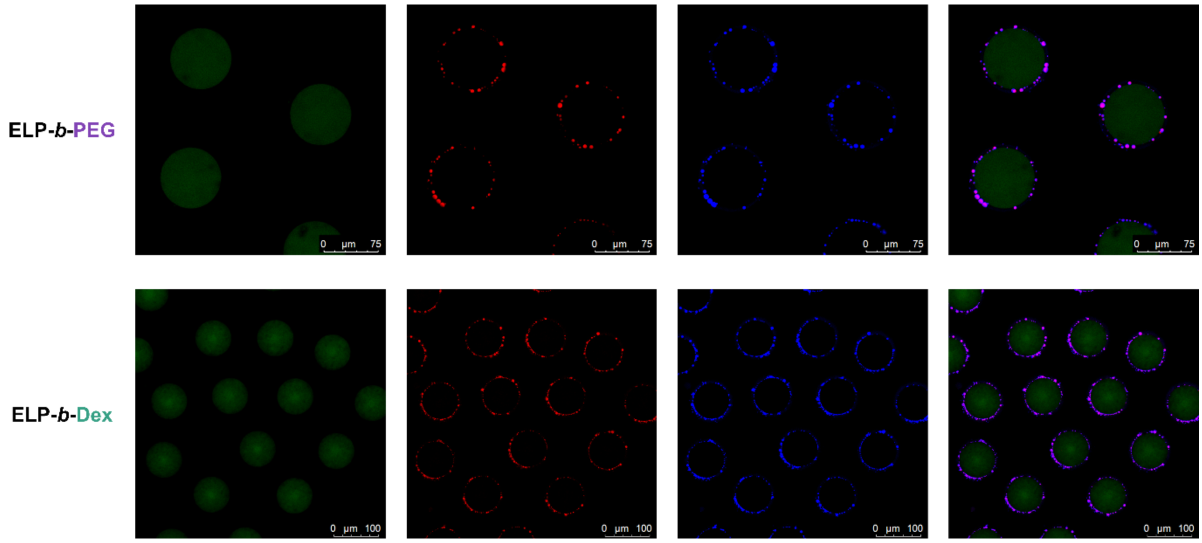


**Figure S13.** Representative confocal images of ELP-polymer stabilized ELP coacervates at 50 °C showing co-assembly of surfactant-like ELP-polymer conjugates with monoblock ELP and the dynamic distribution of these architectures toward the interface between dextran/PEG system.

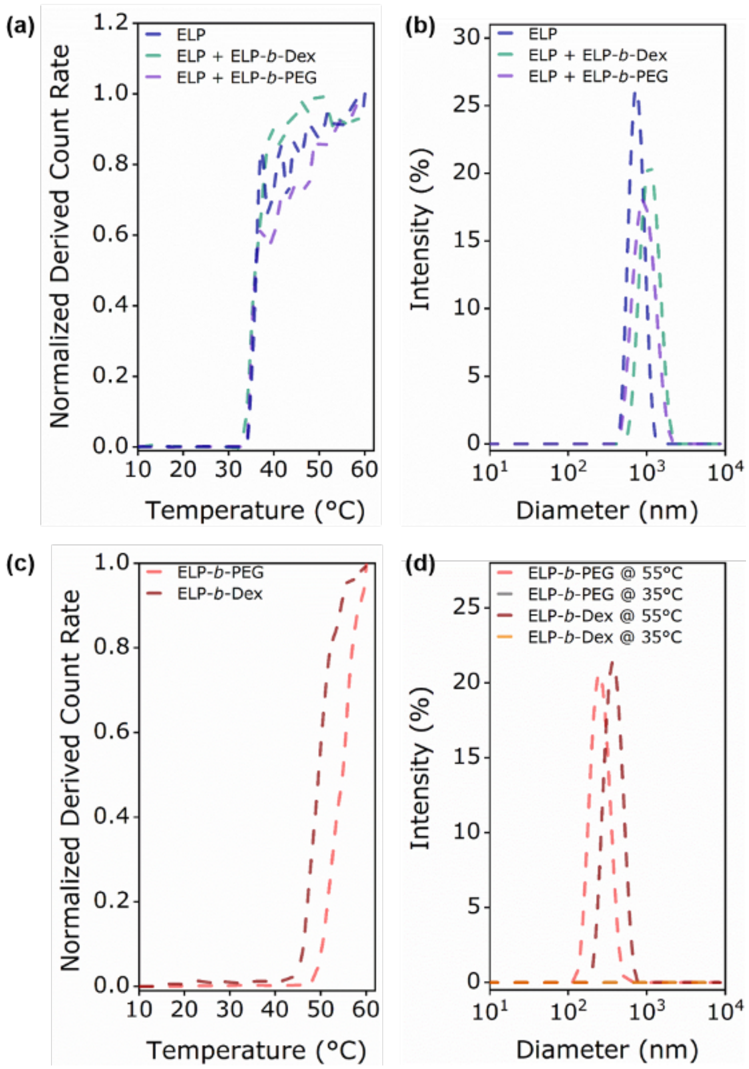


**Figure S14.** Study of co-assembly of the monoblock ELP with ELP-polymer conjugates in pure water. (a) Determination of transition temperatures from DLS measurement of three systems in pure water with operating temperature from 10 to 60 °C, namely 0.25 mg $mL^{-1}$ ELP[$M_1V_3$-60], 0.25 mg $mL^{-1}$ ELP[$M_1V_3$-60] + 0.125 mg $mL^{-1}$ ELP-*b*-Dex and 0.25 mg $mL^{-1}$ ELP[$M_1V_3$-60] + 0.125 mg $mL^{-1}$ ELP-*b*-PEG. All three systems show a sharp phase transition around 33 °C that shifts to a higher temperature compared to that of three systems when solubilized in the PEG solution. It can be attributed to the withdrawal of macromolecules such as PEG that can also absorb water molecules results in a decreased hydrophobicity the ELP. (b) DLS analysis of three system in pure water above the transition temperature at 35 °C. (c) Determination of transition temperatures from DLS measurement of each ELP-polymer bioconjugates at 0.125 mg $mL^{-1}$ in pure water with operating temperature from 10 to 60 °C,

which undergo phase transition over 45 °C that is higher than that of the monoblock ELP. (d) DLS analysis of two individual ELP-polymer (0.125 mg $mL^{-1}$ in pure water) at 35 and 55 °C, respectively.

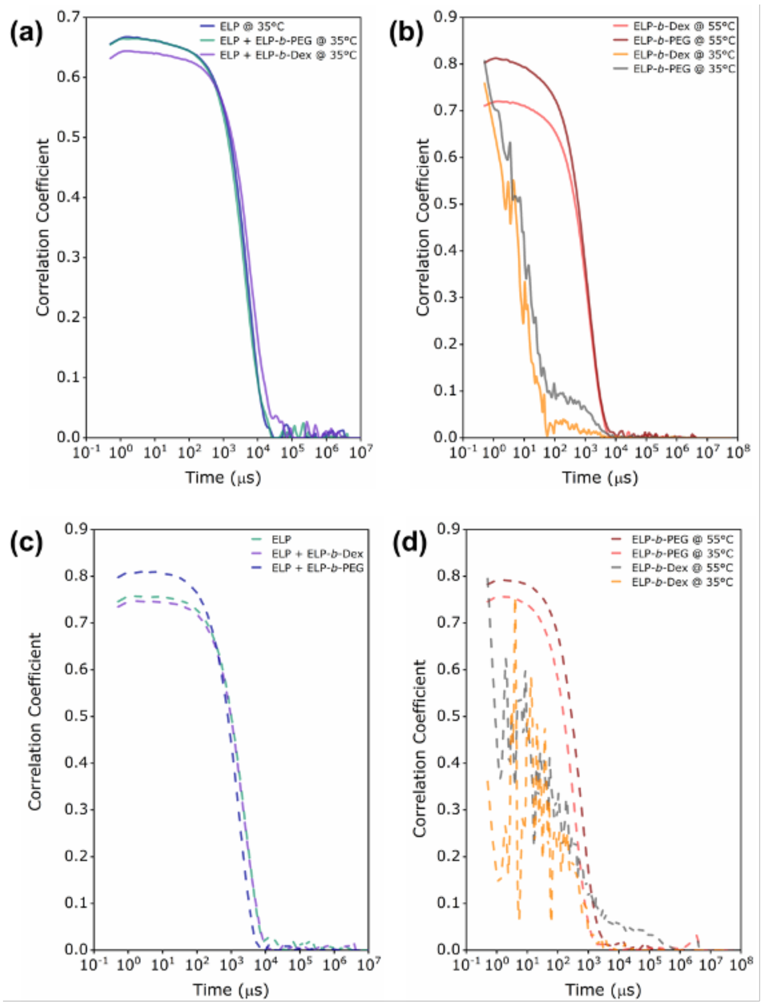


**Figure S15.** DLS intensity autocorrelation function for both systems. (a) and (b) are mixtures dissolved in 8 wt% PEG solution which correlate to Figure 6g and 6h, respectively; (c) and (d) are mixtures dissolved in pure water which correlate to Figure S14b and S14d, respectively.

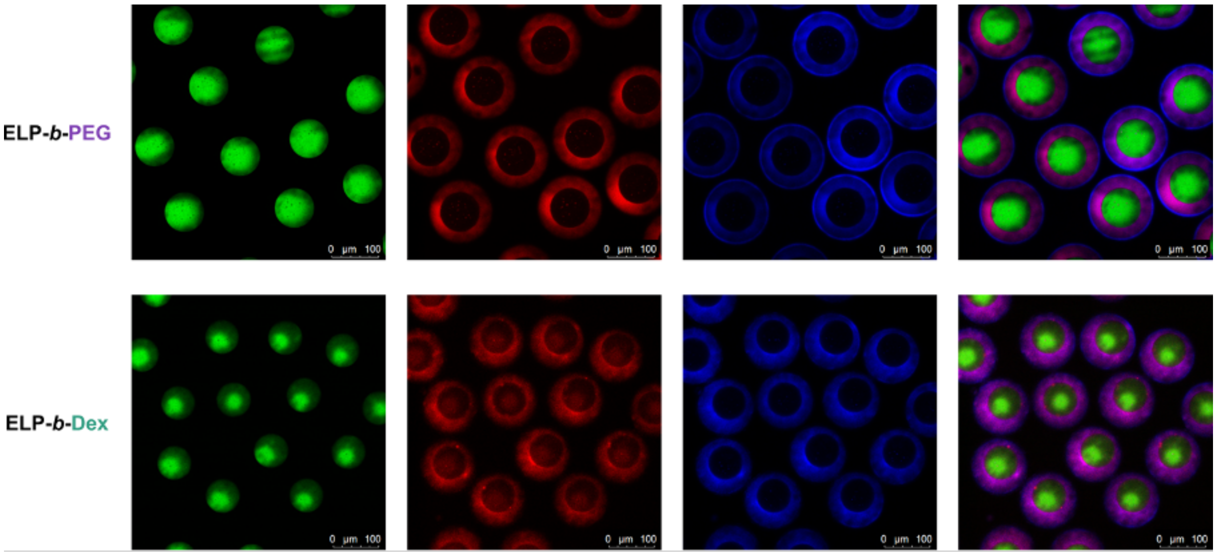


**Figure S16.** Representative confocal images of Rh-ELP-polymer, BDP-ELP and ATPS within microdroplets at 10 °C (below $T_{cp}$) showing thermally induced reversibly dynamic disassembly of compartmentalized ELP coacervates by ELP-polymer conjugates and the spatial organization of the ELP-polymers and the monoblock ELP.

**References**


[1] A. V. Quaethem, P. Lussis, D. A. Leigh, A. S. Duwez, C. A. Fustin, *Chem. Sci.* **2014**, *5*, 1449.

[2] J. Schindelin, I. Arganda-Carreras, E. Frise, V. Kaynig, M. Longair, T. Pietzsch, S. Preibisch, C. Rueden, S. Saalfeld, B. Schmid, *Nat. Methods* **2012**, *9*, 676.

[3] H. Zhao, V. Ibrahimova, E. Garanger, S. Lecommandoux, *Angew. Chem. Int. Ed.* **2020**, *59*, 11028.
